# Metabolite profiling and *in vitro* biological activities of an endophytic *Cladosporium* sp. AUMC 15471 isolated from *Origanum syriacum L*

**DOI:** 10.3389/fphar.2026.1950031

**Published:** 2026-10-23

**Authors:** Mohammed Alaidarous, Ahmed Abdel-Hadi, Abdulrahman Alatiq, Yahya Madkhali, Saeed Banawas, Bader Alshehri, Mohamed E. Abouelela, Yosra A. Helmy, Mohamed Awad, Abdallah Hassane

**Affiliations:** 1 Department of Medical Laboratory Sciences, College of Applied Medical Sciences, Majmaah University, Al Majma’ah, Saudi Arabia; 2 Department of Botany and Microbiology, Faculty of Science, Al-Azhar University, Assiut Branch, Assiut, Egypt; 3 Department of Pharmacognosy, Faculty of Pharmacy (Boys), Al-Azhar University, Cairo, Egypt; 4 Department of Veterinary Science, Martin-Gatton College of Agriculture, Food and Environment, University of Kentucky, Lexington, KY, United States; 5 Department of Biology, College of Science, Taif University, Taif, Saudi Arabia

**Keywords:** biological activity, *Cladosporium*, endophytic fungi, metabolite analysis, *Origanum syriacum*

## Abstract

**Background:**

Endophytic fungi associated with the za’atar plant (*O. syriacum L.*) remain largely underexplored despite their potential as sources of bioactive natural products.

**Objective:**

This study aimed to characterize the endophytic fungus *Cladosporium* sp. AUMC 15471 recovered from *Origanum syriacum* leaves and to investigate its chemical profile and biological activities.

**Methods:**

Identification of *Cladosporium* sp. was achieved by morphological and molecular approaches. Comprehensive metabolite profiling of the ethyl acetate (EtOAc) crude extract was conducted using complementary chromatographic and mass spectrometric techniques, including gas chromatography–mass spectrometry (GC–MS), high-performance liquid chromatography (HPLC), and liquid chromatography–tandem mass spectrometry (LC–MS/MS).

**Results:**

GC–MS analysis tentatively annotated 32 volatile and semi-volatile compounds based on mass spectral database matching and retention index comparison, predominantly monoterpenes, sesquiterpenes, and fatty alcohols. Targeted LC–MS/MS analysis detected 11 metabolites that were assigned based on retention time and mass-spectrometric characteristics in comparison with authentic reference standards. Several of these metabolites have not previously been reported in an endophytic *Cladosporium sp.* associated with *O. syriacum*. The EtOAc crude extract exhibited moderate antioxidant activity in the DPPH assay (IC_50_ = 230.21 ± 5.96 μg/mL). It also showed low cytotoxic activity against HepG2 hepatocellular carcinoma cells (IC_50_ = 93.43 μg/mL), with cell death involving both apoptotic and necrotic pathways. In addition, the extract displayed antibacterial activity against six bacterial species comprising five foodborne bacterial pathogens and one an opportunistic equine pathogen, with inhibition rates of 67.37% ± 2.45% against *Staphylococcus aureus* and 59.39% ± 1.24% against *Campylobacter jejuni*.

**Conclusion:**

Collectively, these findings indicate that *Cladosporium* sp. associated with *O. syriacum* is a promising source of chemically diverse metabolites with potential biological activities.

## 1 Introduction

Endophytes, advantageous microorganisms residing within plant tissues without causing harm and deriving shelter and nourishment (Hyde et al., 2019); have attracted interest for their capacity to enhance plant growth and resilience to stress. Bioactive compounds generated by endophytes enhance antioxidant defenses (Poveda et al., 2020). Endophytic fungi possess the capability to produce therapeutic biologically active compounds with several unexplored pharmacological properties, rendering them a promising novel source of bioactive substances with therapeutic promise for various disorders in the pharmaceutical sector (Adione et al., 2022). The isolation and cultivation of endophytes, along with the purification and characterization of their natural products, have resulted in the discovery of novel antibiotics, anti-mycotics, immunosuppressant, and anticancer agents (Pillai and Karunagaran, 2025).

*Origanum* species have been employed as spices and in ethnomedicine for millennia. Many herbs are utilized in homeopathic, complementary, and conventional medicine due to their antioxidant, antibacterial, anticancer, and other properties. The biological characteristics render *Origanum* species active herbs (de Souza et al., 2006; Mechergui et al., 2010). *Origanum syriacum*, commonly referred to as za’atar, is a prevalent medicinal herb belonging to the Lamiaceae family. It grows extensively in the Middle East and exhibits a variety of morphological and chemical characteristics. Earlier research has identified bioactive metabolites and *in vitro* pharmacological characteristics of herbs from diverse geographical regions, including Palestine, Jordan, Syria, Lebanon, Egypt, and Libya (Alwafa et al., 2021). Phenolic compounds, carotenoids, thymol, carvacrol, thymoquinone, rosmarinic acid, ursolic acid, and flavone glycosides, which possess antioxidant, anti-inflammatory, anticancer, anti-bacterial, and neuroprotective activities have been identified from *O. syriacum* (Alma et al., 2003; Alonazi et al., 2021). Folk medicine employs *O. syriacum* for dermatological fungal infections, gastrointestinal discomfort, pharyngeal infections, cough, and as an anti-inflammatory agent (Abdel-Kader et al., 2018). *Origanum syriacum* suppressed THP-1 human leukemia cells, LoVo, SW620, HCT 116, Vero, and MCF-7 breast cancer cells (AlKahlout et al., 2022).

The fungus genus *Cladosporium* is classified within the Dematiaceae family, under the Dothideomycetes order of Ascomycota (Li, 2013). *Cladosporium* species has been documented in several environmental settings, including air, soil, water, plant and animal life (Li et al., 2024). Moreover, it is present in unfavorable environments, including deserts, highly saline areas, and soils contaminated by radioactivity (Segers et al., 2015). Certain species produce mycotoxins that function as infections in humans as well as animals (Sandoval-Denis et al., 2016). Consequently, numerous species are examined for their biotechnological potential owing to the production of metabolites, including antibiotics, organic acids, and enzymes (Ai et al., 2015). Endophytic *Cladosporium* species are significant in ecological and functional capacities, facilitating plant growth and improving nutrient-related processes (Choudhary et al., 2023). They also facilitate natural disease suppression by producing bioactive antibacterial and antifungal metabolites (Yehia et al., 2020). It has been reported that *Cladosporium* sp. (AC-1) isolated can produce plant-specific compounds (organic acids and alkaloids) and has a close relationship with the plant metabolism (Hernández-Tasco et al., 2022).

Reactive oxygen species (ROS) are widely recognized for their capacity to induce significant health issues, including cancer. Antioxidants play a critical role in mitigating the impact of ROS (Koldaş et al., 2015), while pathogenic bacteria that contaminate foods are the most prevalent cause of gastrointestinal infections and mortality worldwide (Elmonir et al., 2018). In parallel, the search for a novel lead compounds with a potential activity to target the different cellular machineries of the tumor cells and bacterial activity is the challenges. Fungal secondary metabolites, particularly phenolic and quinone metabolites, have demonstrated antioxidant, antibacterial, and cytotoxic properties, prompting further investigation of fungal sources for these bioactivities (Verma et al., 2022). Accordingly, this study aimed to characterize the secondary metabolites produced by the endophytic fungus *Cladosporium* sp. isolated from *O*. *syriacum* and to assess the antioxidant, antibacterial, and cytotoxic activities of its extract.

## 2 Materials and methods

### 2.1 Fungal isolation, identification, cultivation, and extraction

The endophytic fungus *Cladosporium* sp. (Fresen.) G.A. de Vries used in this study was recovered from healthy leaves of *O. syriacum* collected from a commercial nursery in Assiut, Egypt. Seeds of *O. syriacum* were obtained from the Agricultural Research Center, Giza, Egypt, and cultivated at the nursery of the Agricultural Research Center, Assiut, Al-Azhar University, under the supervision of the Department of Horticulture, Faculty of Agriculture, Al-Azhar University, Assiut, Egypt. A voucher specimen of the plant was deposited in the herbarium of the Botany and Microbiology Department, Faculty of Science, Al-Azhar University, Assiut, Egypt, under voucher number ABH361-194.51. Plant samples were collected in July 2025 for subsequent isolation and characterization of endophytic fungi. Following the procedure described by Kumar et al. (2011), endophytic fungi were isolated from surface-sterilized leaves of *O*. *syriacum*. Briefly, five independent *O. syriacum* plants were sampled, and three leaves were collected from each plant, resulting in a total of 15 leaves processed for endophytic fungal isolation. the leaves were initially washed with sterile distilled water to remove surface debris and dust and then cut into small segments using a sterile scalpel. The segments were immersed in 70% ethanol for 30 s, followed by treatment with 1% sodium hypochlorite for 2 min, and subsequently rinsed three times with sterile distilled water. Following surface sterilization, the leaves were aseptically processed and placed on potato dextrose agar (PDA) medium containing potato infusion (200 g/L), dextrose (20 g/L), and agar (20 g/L), with the pH adjusted to 6.5, and incubated under appropriate conditions cultured for fungal isolation. The final washing solution was also cultured as a sterility control, and no fungal growth was observed, supporting the effectiveness of the surface sterilization procedure. Fungal hyphae emerging from the internal tissues of the leaf segments were aseptically transferred to fresh PDA plates and subcultured until pure cultures were obtained. The isolates were initially characterized based on macroscopic and microscopic morphological features and maintained on PDA slants at 4 °C until further analysis. The fungal isolate was subjected to molecular identification following the procedures described by Mohamed et al. (2020) and Abdelrahem et al. (2024). Genomic DNA was extracted and used as a template for PCR amplification of the internal transcribed spacer (ITS) region using the universal primers ITS1 (5′-TCC GTA GGT GAA CCT GCG G-3′) and ITS4 (5′-TCC TCC GCT TAT TGA TAT GC-3′). The resulting PCR products were purified and sequenced, and the obtained sequences were compared with reference sequences available in the NCBI GenBank database using the BLAST algorithm (http://www//ncbi.nlm.nih.gov/BLAST/, accessed on 13 May 2026). The fungal isolate was deposited in the Assiut University Mycological Centre (AUMC) culture collection under accession number AUMC 15471. The resulting ITS sequence was deposited in GenBank under accession number (OM854799) and had a total length of 533 bp. The obtained sequence was aligned with ITS sequences of closely related taxa retrieved from GenBank using BLAST search tool at the NCBI database.

For secondary-metabolite production, a spore suspension (approximately 1 × 10^7^ spores/mL) was prepared from a 7-day-old PDA culture and inoculated into sterilized rice medium. Five 1-L Erlenmeyer flasks, each containing 100 g of rice and 100 mL of distilled water. The substrate was sterilized by autoclaving at 121 °C for 20 min and subsequently cooled to room temperature before inoculation with the fungal isolate. The substrates were inoculated and incubated under static conditions at 28 °C ± 2 °C for 30 days. Following incubation, each fermented culture was extracted twice with ethyl acetate (500 mL per extraction per flask). Ethyl acetate was selected as the extraction solvent because of its intermediate polarity and ability to recover a broad spectrum of moderately polar fungal secondary metabolites, while facilitating solvent removal and subsequent chemical profiling. The combined organic extracts were filtered and concentrated under reduced pressure at 45 °C using a rotary evaporator. The resulting crude extract (7.88 g in total; equivalent to 15.70 mg extract/g rice) was stored until further chemical and biological analyses, following the extraction procedure described by Al Mousa et al. (2021).

### 2.2 Evaluation of the antibacterial activity against food-borne pathogens

To assess the EtOAc crude extracts' antibacterial efficacy, we tested their effect on the growth of a six bacterial species comprising five foodborne bacterial pathogens (*Escherichia coli O157, S. enterica* serovar *Typhimurium, StaphylococcusS. aureus, Campylobacter jejuni* and *Listeria monocytogenes*) and an opportunistic equine pathogen (*Rhodococcus equi*), as previously described (Helmy et al., 2018). The antibacterial assay was performed using a final extract concentration of 1.5 mg/mL in each well. Extract stock solutions were prepared at 150 mg/mL. The final concentration of DMSO in the assay wells was 0.01%, which was confirmed to have no detectable antimicrobial activity. For each bacterial strain, two independent biological replicates were conducted, with each replicate analyzed in duplicate wells. Antibacterial activity was expressed as percentage growth inhibition and calculated according to the following equation: [(OD_600_ of DMSO-treated control - OD_600_ of extract-treated sample)/OD_600_ of DMSO-treated control] × 100. Before calculating percentage inhibition, the initial OD_600_ values of all treatments were corrected by subtracting the OD_600_ of the corresponding uninoculated culture medium (negative control) to eliminate background absorbance, as described by Kumar et al. (2016). The incubation conditions for each bacterial strain were as follows: *E. coli* O157 (ATCC 35150), *Salmonella enterica* serovar *Typhimurium* (ATCC 14028), *Listeria* monocytogenes (ATCC 15313), methicillin-susceptible *Staphylococcus aureus* (MSSA; ATCC 12600), and *R. equi* 103S were incubated for 24 h at 37 °C. *Campylobacter jejuni* 81–176 (ATCC 33560) was incubated for 48 h at 42 °C. For C. jejuni, microaerophilic conditions were maintained throughout the microplate assay using an Anoxomat® Anaerobic Jar System (Nova Biomedicals, MA, United States), with the oxygen concentration adjusted to 6%.

### 2.3 Cell cytotoxicity assay

The cytotoxic activity of the EtOAc crude extract was evaluated against HepG2 (human hepatocellular carcinoma), HeLa (human cervical carcinoma), and SKOV-3 (human ovarian carcinoma) cell lines obtained from Nawah Scientific Inc. (Cairo, Egypt) using the sulforhodamine B (SRB) assay, as previously described by Skehan et al. (1990) and Alsehli et al. (2024), with minor modifications. Briefly, aliquots of cell suspension (100 μL; 5 × 10^3^ cells/well) were seeded into 96-well plates and incubated in complete culture medium. After 72 h incubation, the cells were then treated with 100 µL of medium containing the fungal extract at the indicated concentrations. For preliminary cytotoxicity screening, HepG2, HeLa, and SKOV-3 cells were treated with the fungal extract at 10 and 100 μg/mL. Based on the preliminary screening results, HepG2, the most responsive cancer cell line, and BNL normal mouse liver cells were subsequently exposed to serial concentrations of the fungal extract ranging from 0.01 to 100 μg/mL for IC_50_ determination. Doxorubicin was tested in parallel as the reference drug. Following treatment, the culture medium was removed, and the cells were fixed by adding 150 µL of 10% trichloroacetic acid (TCA) per well and incubating the plates at 4 °C for 1 h. The TCA solution was then removed, and the plates were washed five times with distilled water. Subsequently, 70 µL of SRB solution (0.4%, w/v) was added to each well, and the plates were incubated in the dark at room temperature for 10 min. Unbound dye was removed by washing the plates three times with 1% acetic acid, after which the plates were allowed to air-dry overnight. Protein-bound SRB was solubilized by adding 150 µL of 10 mM Tris solution to each well, and absorbance was measured at 540 nm using an Infinite F50 microplate reader (TECAN, Switzerland). Cell viability was calculated.

### 2.4 Effect of fungal extract on HepG2 cell cycle distribution and assessment of apoptosis

To investigate the mechanism underlying the cytotoxic activity of the EtOAc crude extract, HepG2 cells were treated with the extract at its IC_50_ concentration (IC_50_ = 93.43 μg/mL). for 48 h and subsequently analyzed for cell cycle progression and apoptosis as previously described (Bashmail et al., 2018; Fekry et al., 2019; Al Mousa et al., 2022; Fayed et al., 2022). After treatment with the test compounds for the specified duration, cells (1 × 10^5^ cells) were collected by trypsinization and washed twice with ice-cold PBS (pH 7.4). The cells were resuspended in 2 mL of 60% ice-cold ethanol and incubated at 4 °C for 1 h for fixation. The fixed cells were subsequently washed twice with PBS (pH 7.4) and resuspended in 1 mL of PBS containing 50 μg/mL RNase A and 10 μg/mL propidium iodide (PI). Following incubation for 20 min in the dark at 37 °C, the cells were analyzed for DNA content by flow cytometry using the FL2 fluorescence detection channel of an ACEA NovoCyte™ flow cytometer (ACEA Biosciences Inc., San Diego, CA, United States). For each sample, 12,000 events were acquired. Cell-cycle distribution was determined using ACEA NovoExpress™ software (ACEA Biosciences Inc., San Diego, CA, United States). Apoptotic and necrotic cell populations were quantified using an Annexin V-FITC apoptosis detection kit (Abcam Inc., Cambridge Science Park, Cambridge, United Kingdom) according to the manufacturer’s protocol, followed by dual-color flow cytometric analysis.

### 2.5 Estimation of total phenolic content

The total phenolic content (TPC) of the EtOAc crude extract was determined using the Folin–Ciocalteu colorimetric method as previously described by Suleria et al. (2012) with slight modifications. Briefly, an A half mL of the extract (10 mg/mL) aliquot was mixed with same volume of Folin–Ciocalteu reagent and allowed to react for 5 min before the addition of 1 mL of 10% Na_2_CO_3_ (AL-Nasr Chemicals Co., Cairo, Egypt). The reaction mixture was incubated in the dark at room temperature for 30 min, after which the absorbance was measured at 750 nm using a UV–Vis spectrophotometer. Gallic acid was used to construct the standard calibration curve, and the results were expressed as milligrams of gallic acid equivalents (mg GAE/g dry extract). All measurements were performed in triplicate, and the values were reported as the mean ± standard deviation. The standard curve of gallic acid was linear between 0.5 and 100 μg/mL with curve equation: y = 0.0169x–0.1172, R^2^ = 0.9588.

### 2.6 Antioxidant activity assays

#### 2.6.1 DPPH scavenging activity assay

Briefly, 1.8 mL of 0.1 mM DPPH (1,1-Diphenyl-2-picryl hydrazyl) solution (Sigma-Aldrich, Germany) was added to 0.2 mL of the fungal extract in absolute methanol at various concentrations (1–0.05 mg/mL). Absorbance was determined by a spectrophotometer (Bibby Scientific Ltd., Stone, Staffordshire, UK) at 517 nm after 30 min (Gulcin, 2020). Trolox (Sigma-Aldrich, Germany) was employed as a positive control at concentrations ranging between 8.75 and 2.5 μg/mL. The capacity to scavenge the DPPH radical and the IC_50_ was calculated using linear regression analysis.

#### 2.6.2 ABTS scavenging assay

The EtOAc crude extract was dissolved in dimethyl sulfoxide (DMSO) (MilliporeSigma—St. Louis, MI, United States) and diluted with methanol to a concentration of 10 mg/mL. Subsequently, five serial dilutions were prepared at final concentrations of 7.81–125 μg/mL. The assay ABTS scavenging capacity was conducted in accordance Elkholy et al. (2023). A microplate reader (BMG LABTECH GmbH, Ortenberg, Germany) was employed to measure the decrease in ABTS color intensity at 734 nm at the end of the incubation period. Trolox (Sigma-Aldrich, Germany) was used as standard positive control, at the following final concentrations: 8.75, 6.25, 5, 3.75, and 2.5 μg/mL. The scavenging capacity was determined and the IC_50_ value was calculated.

#### 2.6.3 FRAP assay

The assay was carried out according to the method of Benzie and Strain (1996) with modifications adopted by Elkholy et al. (2023). Briefly, 190 μL from the freshly prepared TPTZ reagent was mixed with 10 μL of the fungal extract sample in a 96-well plate (n = 3), and the reaction was incubated at room temperature in the dark for 30 min. At the end of the incubation period, the resulting blue color was measured at 593 nm. The results were recorded using a microplate reader, BMG LABTECH®-FLUOstar Omega microplate reader (Ortenberg, Germany). Results of the samples are presented as μg TE/mg sample using the linear regression equation extracted from the Trolox calibration curve (linear dose-response curve of Trolox) y = 0.0077x + 0.2716, R^2^ = 0.9999.

### 2.7 Gas chromatography analysis (GC)

The GC-MS system (Agilent Technologies) was equipped with gas chromatograph (7890B) and mass spectrometer detector (5977A) at Central Laboratories Network, National Research Centre, Cairo, Egypt, following a standard protocol (Mohamed et al., 2023). Identification of different constituents was determined by comparing the spectrum fragmentation pattern with those stored in Wiley and NIST Mass Spectral Library data. For analysis of non-volatile compounds, the sample was resuspended in 50 µL of bis(trimethylsilyl)trifluoroacetamide (BSTFA)+ trimethylchlorosilane (TMCS) 99:1 reagent and 50 µL pyridine for silylation of the non-volatile compounds prior to GC analysis. The GC–MS assignments have also been described as putative identifications based on mass-spectral library matching rather than definitive structural confirmation. To improve the reliability of compound identification, a homologous series of n-alkane standards (C10–C28) was analyzed under the same GC–MS conditions as the samples. Linear retention indices (LRI) were calculated according to the Van den Dool and Kratz equation:

$$\text{LRI}=100\times \left[n+\left(\text{tR}\left(x\right)-\text{tR}\left(n\right)\right)/\left(\text{tR}\left(n+1\right)-\text{tR}\left(n\right)\right)\right]$$

where tR(x) is the retention time of the target compound, and tR(n) and tR(n+1) are the retention times of the n-alkanes eluting immediately before and after the compound, respectively. Compound identification was achieved through comparison of mass spectra with the NIST library together with agreement between calculated and published retention indices.

### 2.8 High-performance liquid chromatography analysis

HPLC analysis was performed using 10 μL of the EtOAc crude extract sample injected into an Agilent 1,260 series system equipped with a Zorbax Eclipse Plus C8 column (4.6 × 250 mm, 5 μm). The mobile phase consisted of water (solvent A) and 0.05% trifluoroacetic acid in acetonitrile (solvent B), delivered at a flow rate of 0.9 mL/min (Hassane et al., 2022a). The gradient elution program was as follows:

**Table:** 

Time (min.)	Water (a,%)	Acetonitrile/trifluoroacetic acid (B, %)
0.0	82	18
1.0	82	18
11.0	75	25
18.0	60	40
22.0	82	18
24.0	82	18

The column temperature was maintained at 40 °C, the injection volume was 5 μL, and detection was performed at 280 nm. External flavonoid and phenolic standards were used for the identification and quantification of compounds. Based on peak area computation, the quantification of compounds was analyzed (Hassane et al., 2022b).

### 2.9 LC-MS analysis

Targeted liquid chromatography–electrospray ionization tandem mass spectrometry (LC-ESI-MS/MS) analysis was performed using an ExionLC™ AC liquid chromatography system (SCIEX, United States) coupled to a SCIEX Triple Quad 5,500+ mass spectrometer (SCIEX, Singapore) equipped with an electrospray ionization (ESI) source. A 5 μL aliquot of each sample was injected for chromatographic separation and analyzed in multiple-reaction monitoring (MRM) mode following the procedure described by Al Mousa et al. (2024). The analysis was targeted toward predefined phenolic and flavonoid compounds for which specific precursor-to-product ion transitions had been established. Authentic reference standards were used for compound assignment and quantification, as appropriate, following Al Mousa et al. (2023). Analyte assignment was based on comparison of retention times and characteristic MRM transitions with those obtained from the corresponding authentic standards analyzed under identical chromatographic and mass-spectrometric conditions. Where quantitative analysis was performed, calibration curves were prepared using a series of standard solutions at different concentrations and were used to determine the concentrations of the corresponding analytes. Method performance was evaluated in terms of linearity, sensitivity, and repeatability. Linearity was assessed using the regression equation and coefficient of determination (R^2^) obtained for each analyte. The limits of detection (LOD) and quantification (LOQ) were calculated according to the ICH approach using the standard deviation of the response (σ) and the slope of the calibration curve (S), according to the equations LOD = 3.3σ/S and LOQ = 10σ/S. Repeatability was evaluated by replicate injections of the same sample and expressed as the percentage relative standard deviation (%RSD). Accordingly, the LC-ESI-MS/MS procedure was used as a targeted analytical method for the predefined analytes and was not intended as an untargeted metabolomics workflow.

### 2.10 Statistical analysis

All experiments were performed as three independent biological replicates (n = 3), each performed on separate days with different cell passages (P5-P15) and fresh extract dilutions. Biological replicate = independent experiment on separate day; technical replicate = 3 wells per concentration within each biological replicate averaged to one biological value. Flow cytometry: 12,000 events per biological replicate. IC_50_ calculated by nonlinear regression (four-parameter logistic, log(inhibitor) vs normalized response - variable slope, GraphPad Prism v9.5.1, Bottom = 0 Top = 100, R^2^ and 95% CI reported). Normality assessed by Shapiro-Wilk (p > 0.05) and QQ plots, variance homogeneity by Levene’s test; Welch’s correction applied where heterogeneous. For n = 3, results confirmed with Mann-Whitney U test (concordant). For two-group comparisons (Tables 1, 2): unpaired two-tailed Welch’s t-test. Multiple t-tests across 4 populations corrected by Holm-Sidak (alpha = 0.05). Significance: ns p ≥ 0.05, *p < 0.05, **p < 0.01, ***p < 0.001, ****p < 0.0001. Exact p-values reported. The antibacterial assay was performed using two independent biological experiments, with duplicate wells for each condition (n = 2 biological replicates; technical duplicate measurements).

**TABLE 1:** Flow cytometric quantification of HepG2 cell populations after 48h treatment.

Group	Viable (%)	Early apoptosis (%)	Late apoptosis (%)	Necrosis (%)
Control	95.82 ± 0.71	1.61 ± 0.10	0.36 ± 0.15	2.20 ± 0.58
*Cladosporium* sp. EtOAc 93.43 μg/mL (IC50)	84.40 ± 0.31 *** p = 6.0 × 10^−5^	0.70 ± 0.08 *** p = 2.0 × 10^−4^	5.98 ± 0.06 **** p < 1 × 10^−5^	8.91 ± 0.21 *** p = 8.0 × 10^−5^

Data are presented as mean ± SD, of three independent biological replicates (n = 3), each performed on separate days with different passages. 10,000 events acquired per replicate for apoptosis (FSC-A, vs SSC-A → FSC-H, vs FSC-A, singlets → FITC-A, vs PE-Texas Red-A). ***p < 0.001, ****p < 0.0001 vs Control (unpaired two-tailed Welch’s t-test, Holm-Sidak correction for 4 comparisons).

**TABLE 2:** Cell cycle distribution of HepG2 cells following 48h treatment with *Cladosporium* sp.

Group	Sub-G1 (%)	G0/G1 (%)	S (%)	G2/M (%)
Control	2.65 ± 1.15	60.79 ± 1.71	19.02 ± 1.77	20.29 ± 1.07
*Cladosporium* sp. 93.43 μg/mL	6.71 ± 0.29 * p = 0.0214	51.88 ± 0.71 ** p = 0.0016	27.48 ± 1.02 ** p = 0.0013	15.44 ± 0.65 ** p = 0.0022

Data are mean ± SD, of three independent biological replicates (n = 3). Sub-G1, indicates apoptotic fraction. *p < 0.05, **p < 0.01 vs Control (unpaired Welch’s t-test, Holm-Sidak correction). Normality confirmed by Shapiro-Wilk p > 0.05, variance by Levene’s test.

## 3 Results

### 3.1 Identification of fungal isolate

A total of 15 fungal isolates were recovered from the 15 processed leaves, comprising four *Cladosporium* isolates, one *Aspergillus niger*, 3 *Aspergillus terreus*, 2 *A. fumigatus*, and 5 *A. chevalieri*. *Cladosporium* was recovered from four independent leaves originating from two different plants, corresponding to an isolation frequency of 26.7% (4/15 leaves). One of the four *Cladosporium* isolates was selected for further investigation based on preliminary biological activity and was subsequently subjected to molecular identification and characterization. The *Cladosporium* isolate was characterized using morphological and molecular approaches by the ITS region sequencing, and sequences (533 nucleotides, Supplementary Material) were subjected to a BLAST search tool at the NCBI database. The fungal colony grown on PDA demonstrated slow growth, distinguished by a olivaceous-brown to black colony and a coarse texture featuring velvety mycelium. Microscopic analysis disclosed sparsely branched conidiophores with small, single-celled, smooth-walled conidia. The isolate was confirmed as *Cladosporium* sp. AUMC 15471 (GenBank accession no. OM854799). It showed 99.44%−100% identity and 98%–100% coverage with several strains of *Cladosporium* sp. including the type strain DM11M1 [MT102827]. Two species of *Alternaria* are included in the tree as outgoup strains (Figure 1).

**FIGURE 1:**
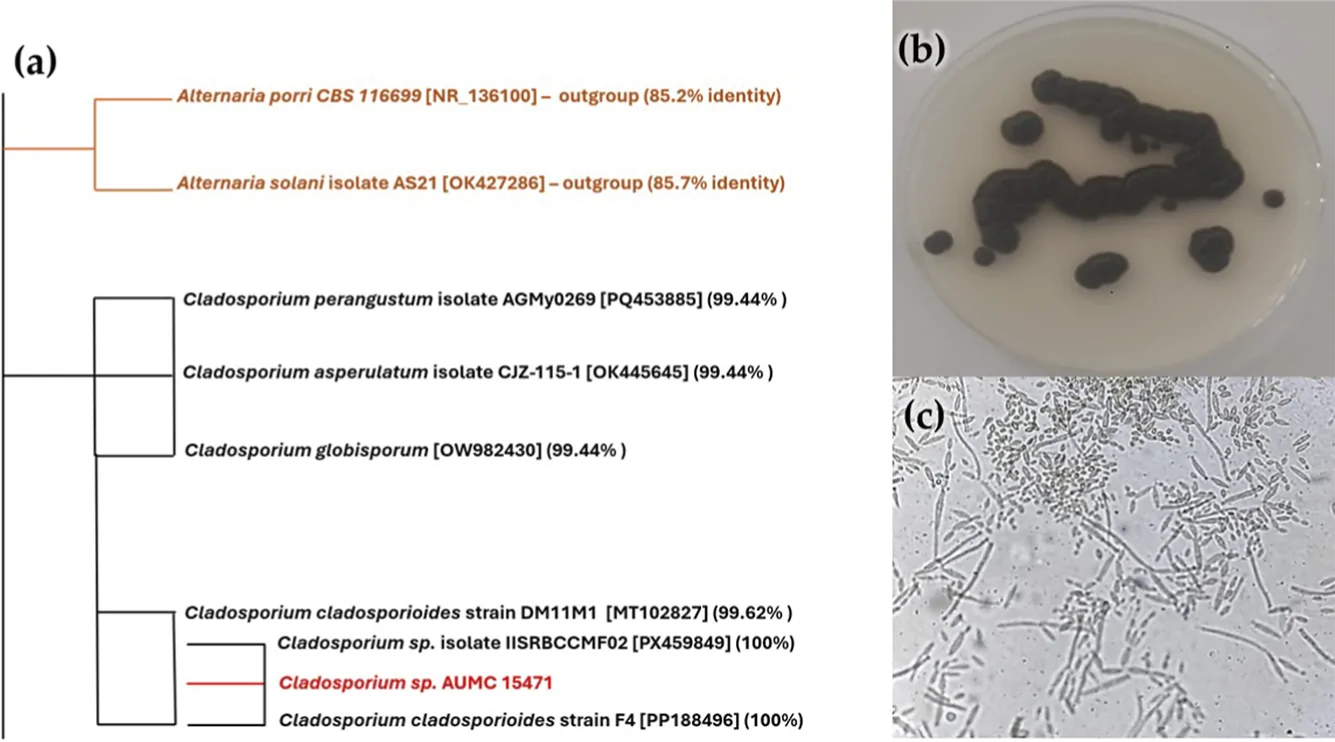
Morphological and molecular characterization of *Cladosporium* sp. AUMC 15471. **(A)** Phylogenetic tree based on ITS rDNA sequences showing the relationship between isolate AUMC 15471 and representative *Cladosporium* species retrieved from GenBank. Sequence accession numbers are shown in parentheses. *Alternaria porri* CBS 116699 (KJ718101) and *Alternaria solani* CBS 107175 (KJ718102) were used as outgroup taxa to root the tree. The tree was constructed using the Maximum Likelihood method in MEGA-X software version 10.1.8 with 1,000 bootstrap replicates. Bootstrap support values above 50% are indicated at the nodes. **(B)** Colony morphology of *Cladosporium* sp. AUMC 15471 grown on PDA. **(C)** Microscopic characteristics showing septate hyphae and conidia.

### 3.2 Antibacterial activity assessment

In the current study, *Cladosporium* sp. EtOAc crude extract was evaluated for its antibacterial effect against *S. Typhimurium*, *Escherichia coli* O157, *Listeria monocytogens*, *S. aureus*, *Rhodococcus equi*, and *C. jejuni* at conc. of 1.5 mg/mL. The EtOAc crude extract exhibited measurable growth inhibition against the tested bacterial strains at the single concentration evaluated (1.5 mg/mL). The highest inhibition was observed against *S. aureus* (67.37% ± 2.45%), followed by *C. jejuni* (59.39% ± 1.24%). Because only one extract concentration was tested, these findings demonstrate growth inhibition under the experimental conditions but do not establish antibacterial potency or permit determination of MIC, MBC, or a concentration–response relationship. Chloramphenicol (20 μg/mL) was included as a positive control; however, its activity was not directly compared quantitatively with that of the crude extract because the two treatments were tested at substantially different concentrations (Figure 2).

**FIGURE 2:**
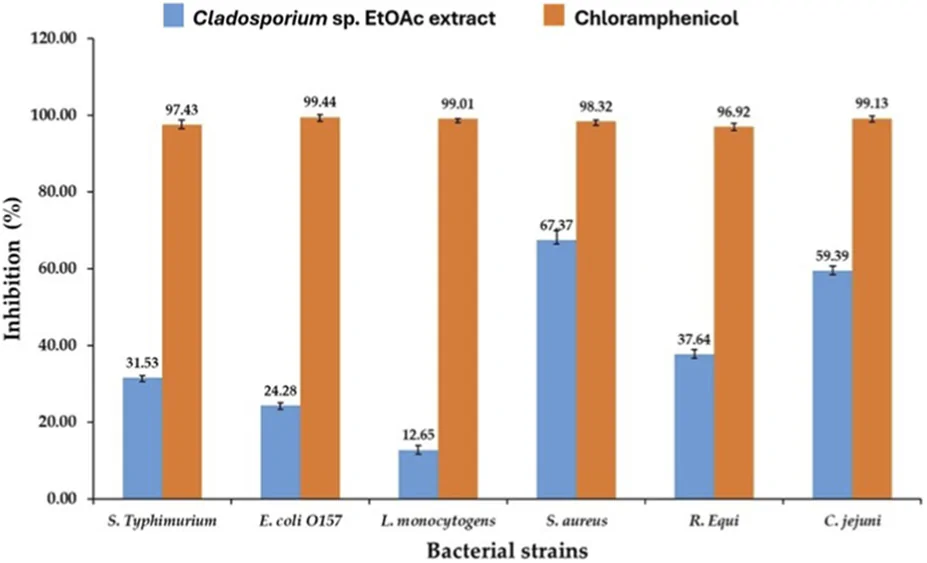
Antibacterial activity of the *Cladosporium* sp. EtOAc crude extract shown as percentage of inhibition at 1.5 mg/mL. Chloramphenicol was tested at 20 μg/mL.

### 3.3 Cytotoxicity assay

The preliminary cytotoxicity quick screening of *Cladosporium* sp. EtOAc crude extract against HepG2, HeLa, and SKOV-3 cell lines at concs. of 10 and 100 μg/mL revealed highest toxicity against HepG2 cell line with viability of 85.87% and 45.81%, respectively (Table 2). Thus, HepG2 cell line was chosen for further IC_50_ determination along with BNL cells.

#### 3.3.1 IC_50_ cytotoxicity determination against BNL and HepG2 cell lines

Cytotoxicity was assessed by sulforhodamine B (SRB) assay. Cells were exposed to five concentrations (0.01, 0.1, 1, 10, 100 μg/mL) plus vehicle control (0.1% DMSO) in triplicate for 72 h (BNL) and 48 h (HepG2) and Doxorubicin (DOX) was used as positive control. Complete concentration-response data, including raw OD values and blank-corrected viabilities, are provided in Supplementary Table S1. For BNL cells, no IC_50_ was reached within the tested concentration range. Viability remained 100.01% ± 1.63% at 0.01 μg/mL, 93.22% ± 2.23% at 0.1 μg/mL, 92.58% ± 1.19% at 1 μg/mL, 90.36% ± 0.91% at 10 μg/mL and 89.42% ± 0.17% at 100 μg/mL (mean ± SD, n = 3). Therefore, IC_50_ is reported as >100 μg/mL. This indicates no significant cytotoxicity up to 100 μg/mL under these conditions tested (Figures 3, 4). On the other hand, *Cladosporium* sp. EtOAc extract exhibited low cytotoxicity and observable reduction in HepG2 cells viability was noticed at concentrations of 100 μg/mL with an IC_50_ value of 93.43 ± 3.06 μg/mL, while the IC_50_ value of 0.14 ± 0.05 μg/mL was provides by the positive standard Doxorubicin (Figures 5, 6). Selectivity was quantified as SI = IC50(normal)/IC50(cancer). Using BNL IC_50_ > 100 μg/mL and HepG2 IC_50_ 93.43 μg/mL, SI = >100/93.43 = >1.07. The obtained SI of >1.07 does not meet the criteria (SI > 3 is generally considered indicative of moderate selectivity and SI > 10 of high selectivity) and indicates lack of meaningful selective cytotoxicity under current test conditions.

**FIGURE 3:**
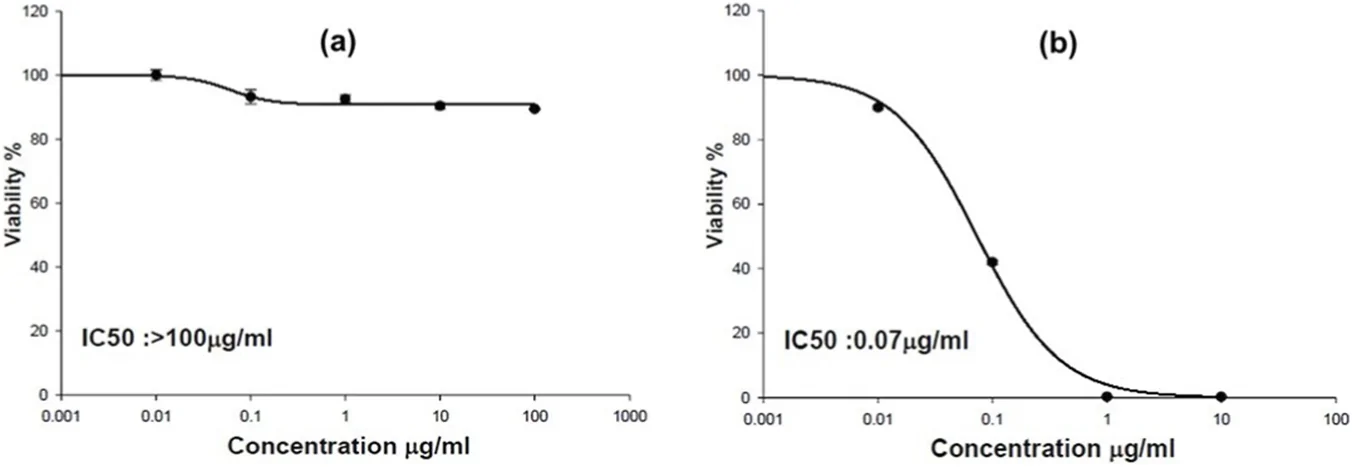
Dose response curve of cytotoxic activity on BNL cells after 72 h treatment **(A)** *Cladosporium* sp. EtOAc crude extract and **(B)** doxorubicin (DOX). Data represent mean ± SD (n = 3).

**FIGURE 4:**
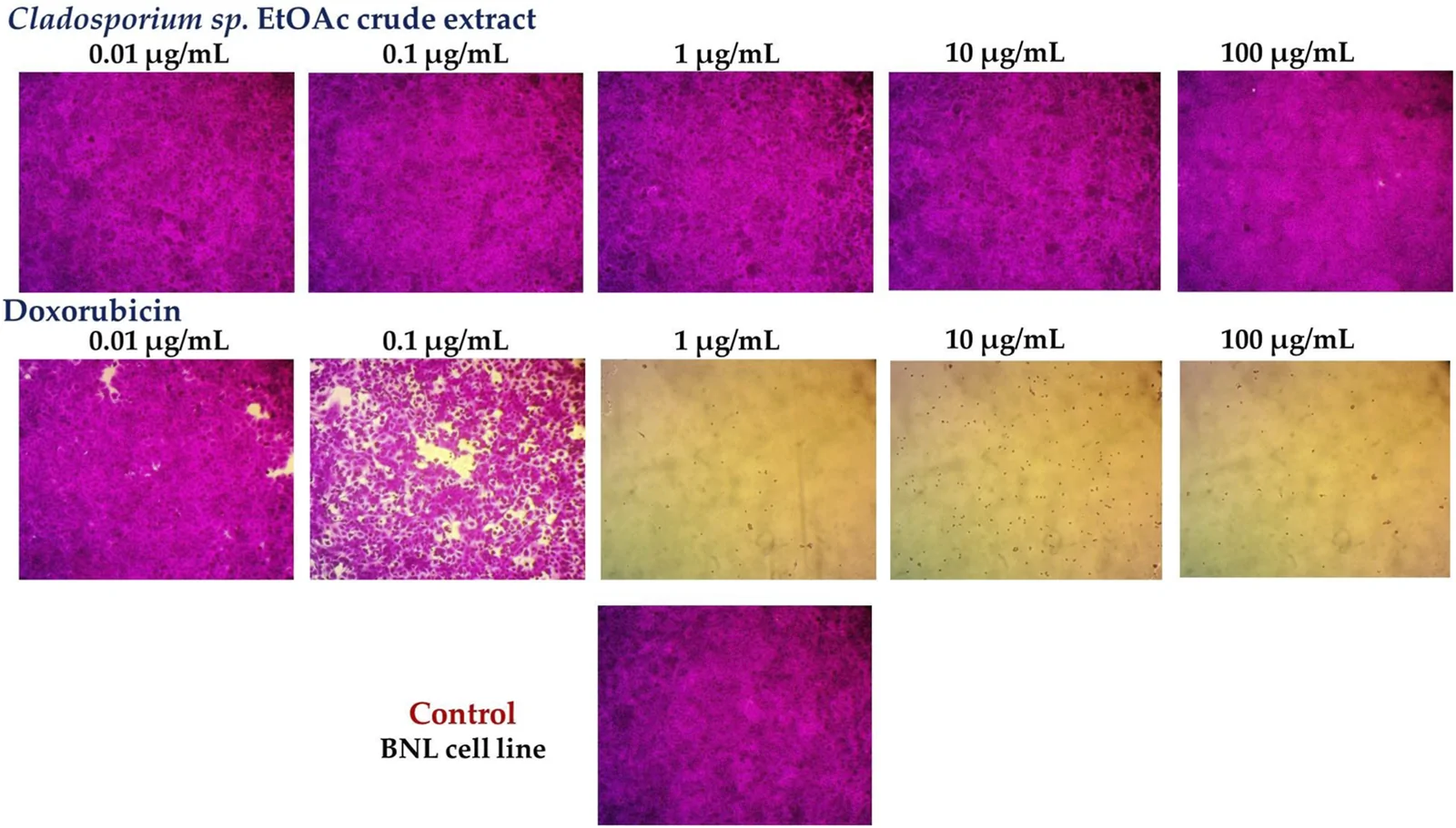
Optical microscope images of cytotoxicity assay of *Cladosporium* sp. EtOAc crude extract and doxorubicin against BNL cell line. Magnification power: ×100.

**FIGURE 5:**
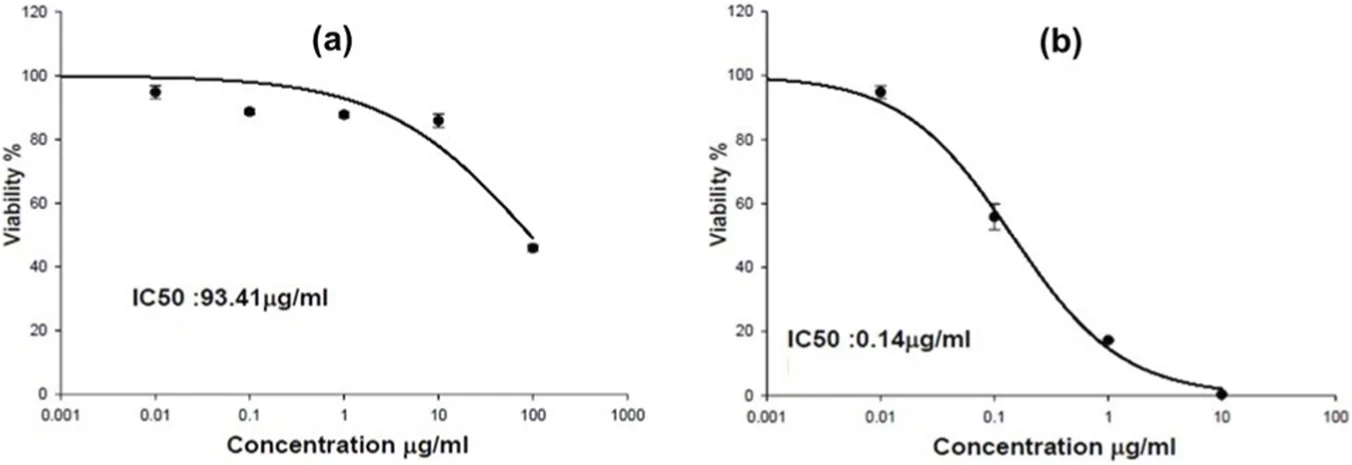
Dose response curve of cytotoxic activity on HepG2 cells after 72 h treatment **(A)** *Cladosporium* sp. EtOAc crude extract and **(B)** doxorubicin (DOX). Data represent mean ± SD (n = 3).

**FIGURE 6:**
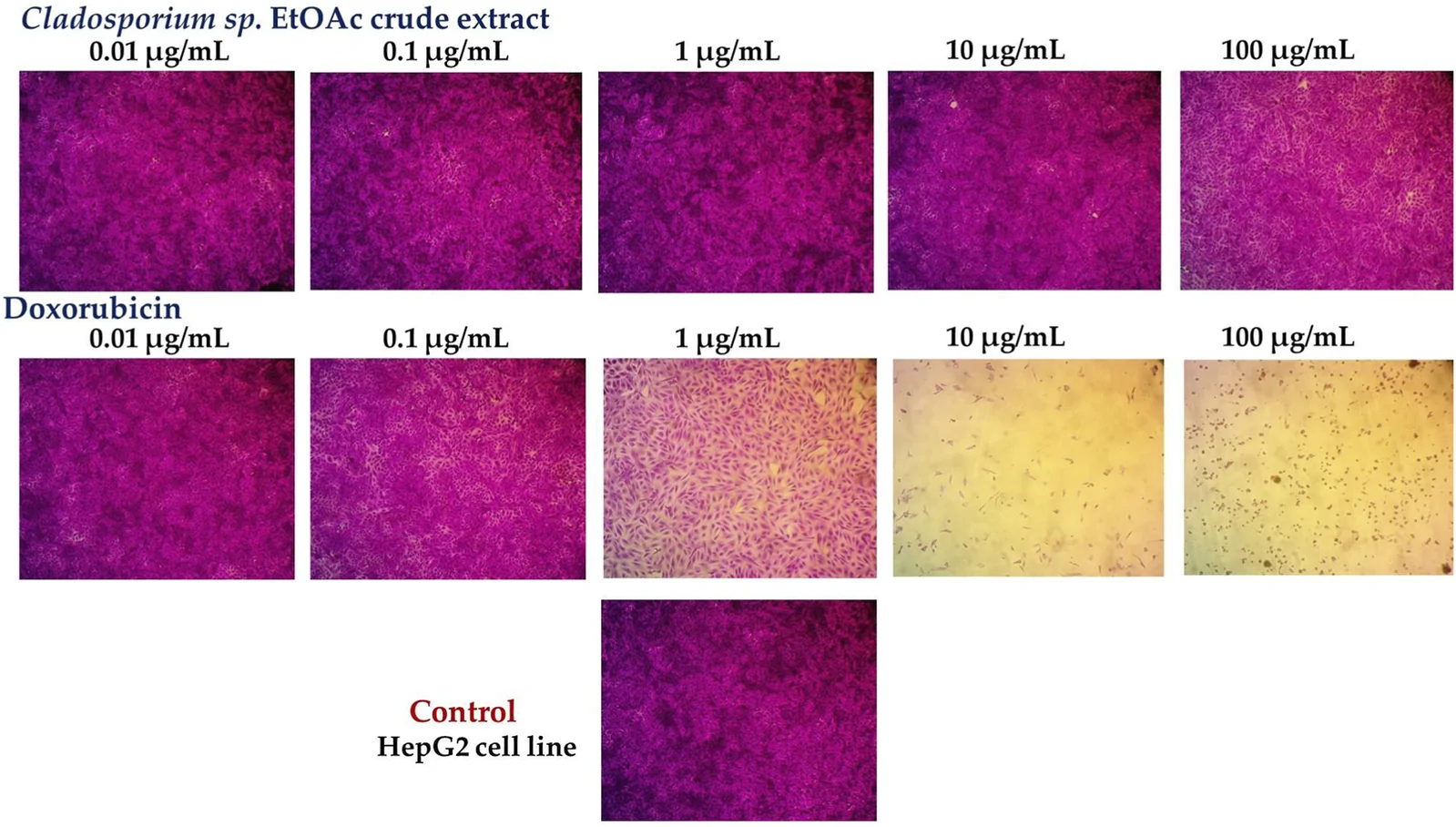
Optical microscope images of cytotoxicity assay of *Cladosporium* sp. EtOAc crude extract and doxorubicin against HepG2 cell line. Magnification power: ×100.

#### 3.3.2 Assessment of apoptosis and HepG2 cell cycle distribution

##### 3.3.2.1 Assessment of apoptosis and necrosis in HepG2 cells

To characterize the distribution of cell populations associated with apoptosis and necrosis following treatment with *Cladosporium* sp. EtOAc crude extract (1C), HepG2 cells were subjected to Annexin V-FITC/PI double staining followed by flow-cytometric analysis after 48 h of treatment. The gating strategy involved an initial selection of the main cell population (P3) based on forward- and side-scatter characteristics to exclude cellular debris, followed by quadrant analysis based on Annexin V and PI staining to distinguish viable, early apoptotic, late apoptotic, and necrotic cell populations. The untreated control cells exhibited a high proportion of viable cells, with 95.82% ± 0.71% of cells located in the viable quadrant (Q3) (Table 3; Figure 7). Following 48 h of treatment with *Cladosporium* sp. EtOAc crude extract, the viable population decreased to 84.40% ± 0.31%. This reduction was accompanied by increases in the necrotic and late apoptotic populations. The necrotic fraction increased from 2.20% ± 0.58% in the control to 8.91% ± 0.21% in the treated cells, while the late apoptotic population (Q2) increased from 0.36% ± 0.15% to 5.98% ± 0.06%. In contrast, the early apoptotic population (Q4) decreased from 1.61% ± 0.10% in the control to 0.70% ± 0.08% following treatment. Thus, at the 48-h time point, the extract was associated with an increased proportion of late apoptotic and necrotic cells, whereas an increase in the early apoptotic population was not observed. Although the lower early apoptotic fraction could be consistent with several possible cellular responses, including differences in the timing of apoptotic events, a temporal progression from early to late apoptosis cannot be established from a single time point. Time-course experiments would therefore be required to determine the kinetics and sequence of these cell-death responses.

**TABLE 3:** Cytotoxicity effect of *Cladosporium* sp. EtOAc crude extract on HepG2, HeLa, and SKOV-3 cell lines.

Tested agent (Cell lines/Concentrations)	Viability %					
	HepG2		HeLa		SKOV-3	
	100 μg/mL	10 μg/mL	100 μg/mL	10 μg/mL	100 μg/mL	10 μg/mL
EtOAc extract	45.81 ± 1.05	85.87 ± 2.13	92.37 ± 0.54	103.71 ± 0.78	95.66 ± 0.41	99.17 ± 0.97
Doxorubicin	1.41 ± 0.16	0.32 ± 0.04	0.66 ± 0.41	5.04 ± 0.74	3.09 ± 0.36	15.37 ± 0.63

Data are presented as mean ± standard deviation (SD) of three independent replicates (n = 3).

**FIGURE 7:**
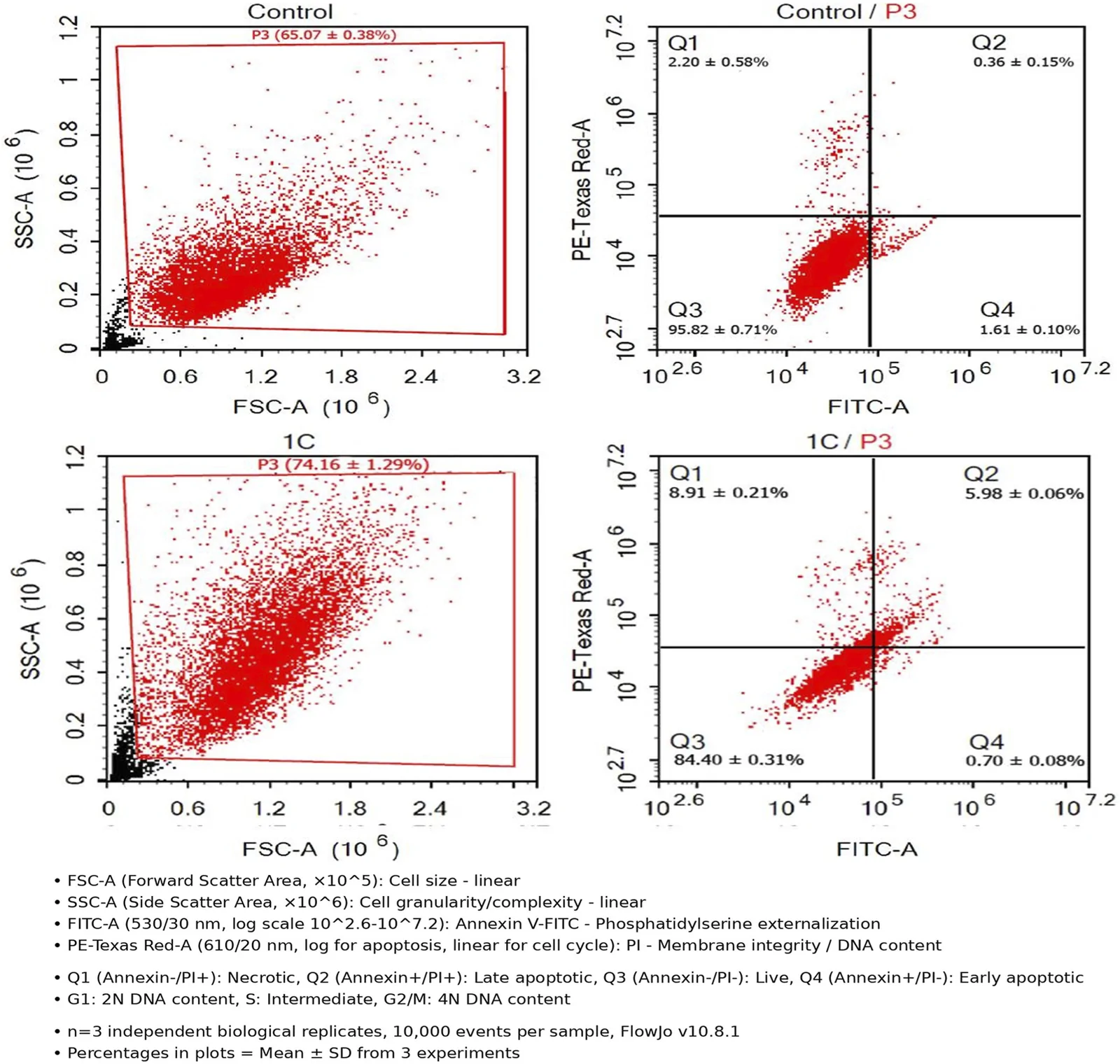
Flow cytometry dot plots showing apoptosis profiles in HepG-2 cells after 48-h treatment with *Cladosporium* sp. (1C) EtOAc crude extract, using Annexin V-FITC/PI staining.

##### 3.3.2.2 Effect of *Cladosporium* sp. extract on cell cycle progression

To further characterize the effects of *Cladosporium* sp. EtOAc crude extract (Sample 1C) on HepG2 cells, cell-cycle distribution was analyzed after 48 h of treatment using propidium iodide (PI) staining followed by flow cytometry. DNA-content histograms were analyzed to determine the proportions of cells in the G_0_/G_1_, S, and G_2_/M phases, while the sub-G1 population was evaluated as an indicator of cells with reduced DNA content, consistent with DNA fragmentation. As shown in Table 1 and Figure 8, untreated HepG2 cells exhibited a typical distribution across the cell-cycle phases, with 60.79% ± 1.71% of cells in G_0_/G_1_, 19.02% ± 1.77% in S phase, and 20.29% ± 1.07% in G_2_/M. Following 48 h of treatment with *Cladosporium* sp. EtOAc crude extract, the cell-cycle distribution was altered, with the S-phase population increasing to 27.48% ± 1.02%. This increase was accompanied by decreases in the G_0_/G_1_ and G_2_/M populations, which decreased to 51.88% ± 0.71% and 15.44% ± 0.65%, respectively. The increased proportion of cells in S phase indicates S-phase accumulation following treatment; however, the present data alone do not establish a definitive S-phase arrest. In addition, the sub-G1 population increased from 2.65% ± 1.15% in the control to 6.71% ± 0.29% in the treated cells. This increase is consistent with enhanced DNA fragmentation and provides supportive evidence of cell death. However, sub-G1 accumulation alone cannot be considered definitive evidence of apoptosis. Taken together with the Annexin V/PI findings, which showed increased late apoptotic and necrotic populations at 48 h, the cell-cycle data indicate that treatment was associated with altered cell-cycle distribution and increased DNA fragmentation, while further time-course studies and complementary molecular markers would be required to establish the underlying mechanism.

**FIGURE 8:**
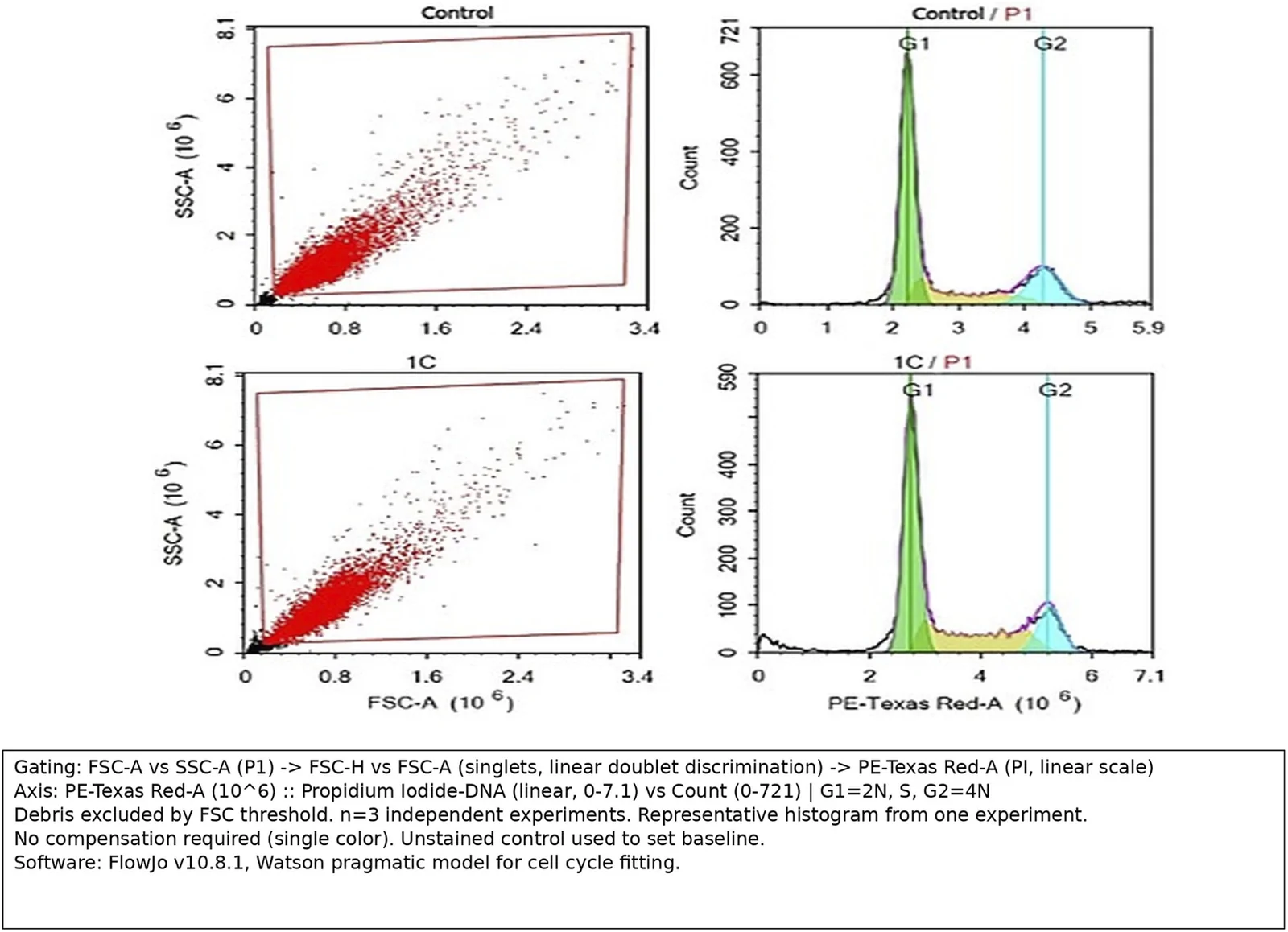
Representative cell cycle histograms of HepG-2 cell lines after 48 h treatment with *Cladosporium* sp. EtOAc crude extract (1C). Cell populations in G0/G1, S, and G2/M phases were quantified by flow cytometry. Percentages for each phase are shown. One representative histogram from three independent experiments is presented (n = 3).

### 3.4 Determination of total phenolic content and antioxidant activity assays

Data presented in Table 4 revealed the total content of phenolics (34.09 mg GAE/g) in ethyl acetate extract from endophytic *Cladosporium* sp. Regarding antioxidant activity, *Cladosporium* sp. EtOAc extract exhibited DPPH and ABTS IC50 values of 230.21 and 183.70 μg/mL, respectively, compared to Trolox IC50 values of 6.50 and 4.69 μg/mL, respectively. Moreover, the investigated fungal extract produced 23.61 μg Trolox eq/mg for FRAP assay which is conversely proportion to activity of DPPH and ABTS.

**TABLE 4:** Total phenolic content and antioxidant activities (IC_50_ values) of EtOAc crude extract from endophytic *Cladosporium* sp.

Extract	Total phenolics (mg GAE/g)	DPPH IC_50_ (µg/mL)	ABTS IC_50_ (µg/mL)	FRAP (µg Trolox eq/mg)
*Cladosporium* sp. EtOAc	34.09 ± 0.73	230.21 ± 5.96 **** p < 0.0001 vs Trolox	183.70 ± 1.02 **** p < 0.0001 vs Trolox	23.61 ± 1.38
Trolox (positive control)	-	6.50 ± 0.40	4.69 ± 1.02	-

-, Not tested. Data are mean ± SD, of three independent biological replicates (n = 3) performed on separate days, each with triplicate technical wells averaged to one biological value. IC_50_ calculated by nonlinear regression (log[inhibitor] vs normalized response, variable slope, GraphPad Prism 9.5.1, constraints Bottom = 0 Top = 100). Statistical comparison vs Trolox by unpaired Welch’s t-test. ****p < 0.0001.

### 3.5 GC-MS analysis

GC-MS analysis of unsaponified *Cladosporium* sp. EtOAc crude extract lead to the detection of 32 tentatively annotated compounds based on their based on spectral similarity and relative area percentages. The EtOAc crude extract contained a mixture of monoterpenes/terpenoids, sesquiterpenes/terpenoids, and fatty alcohols. 8-Isopropyl-1-methyl-3-methylenetricyclo[4.4.0.02,7]decane, a sesquiterpene, was present in a high percentage (18.18%), suggesting its prevalence in the sample, followed by phenol, 2-methyl-5-(1-methylethyl)-, a monoterpenoid phenol, with a percentage of 14.95. Cyclohexene, 1-methyl-4-(1-methylethylidene)-, terpinene, cadinol, and cadinene were detected and exist with percentages of 10.92, 10.75, 8.86, and 8.11%, respectively. It is worth notable the detection of 1-octen-3-ol (0.62%) which is known as fungal alcohol and has an earthy type odor (Table 5; Figure 9).

**TABLE 5:** GC-MS analysis of EtOAc crude extract from endophytic *Cladosporium* sp.

Peak	Compound	RT (min)	RI calculated	RI literature*	Molecular formula	Similarity/Match score (%)	Relative abundance (%)	Identification level (MSI)
1	Pinene	10.906	1,051.7	932	119.7	C10H16	0.25	Level 2*
2	Oct-1-en-3-ol	11.006	1,060.9	980	80.9	C8H16O	0.62	Level 2*
3	1-Methyl-4-(propan-2-ylidene)cyclohex-1-ene	11.149	1,074.1	1,088	13.9	C10H16	10.92	Level 2
4	1-Methyl-4-(propan-2-yl)benzene	11.497	1,105.8	1,024	81.8	C10H14	2.93	Level 2*
5	Ocimene	11.854	1,137.4	1,049	88.4	C10H16	1.84	Level 2*
6	Terpinene	12.092	1,158.5	1,059	99.5	C10H16	10.75	Level 2*
7	Non-2-en-1-ol	12.806	1,225.1	1,158	67.1	C9H18O	0.53	Level 2*
8	3,7-Dimethylocta-1,6-dien-3-ol	13.706	1,317.9	1,099	218.9	C10H18O	0.79	Level 2*
9	Dec-2-enal	17.187	1708.8	1,263	445.8	C10H18O	0.45	Level 2*
10	2-Methyl-5-(propan-2-yl)phenol	18.902	1890.7	1,292	598.7	C10H14O	14.95	Level 2*
11	1-Ethenyl-1-methyl-2,4-bis(prop-1-en-2-yl)cyclohexane	19.321	1944	1,488	456	C15H24	0.95	Level 2*
12	Gurjunene	19.626	1983.5	1,409	574.5	C15H24	0.21	Level 2*
13	Caryophyllene	20.015	2034.3	1,418	616.3	C15H24	2.28	Level 2*
14	Humulene	20.678	2,120.7	1,454	666.7	C15H24	4.23	Level 2*
15	Muurolene	20.873	2,145.5	1,480	665.5	C15H24	0.18	Level 2*
16	8-(Propan-2-yl)-1-methyl-3-methylenetricyclo[4.4.0.0^2^,^7^]decane	21.188	2,185.7	1,376	809.7	C15H24	18.18	Level 2*
17	Elemene	21.44	2,220	1,391	829	C15H24	0.93	Level 2*
18	Cadinene	21.645	2,249.3	1,522	727.3	C15H24	8.11	Level 2*
19	4-(Propan-2-yl)-3,7-dimethyloctahydro-1H-cyclopenta[1,3]cyclopropa[1,2]benzen-3-ol	21.759	2,265.6	1,576	689.6	C15H26O	0.36	Level 2*
20	Cubebol	22.23	2,332.9	1,515	817.9	C15H26O	0.29	Level 2*
21	Epicubebol	23.273	2,495.5	1,533	962.5	C15H26O	0.69	Level 2*
22	Cubenol	23.997	2,616.2	1,646	970.2	C15H26O	0.56	Level 2*
23	Cubedol	24.164	2,644	1,590	1,054	C15H26O	0.34	Level 2*
24	Tau-cadinol	24.326	2,671	1,639	1,032	C15H26O	2.95	Level 2*
25	1,2,3,4,4a,7,8,8a-Octahydro-1,6-dimethyl-4-(propan-2-yl)naphthalen-1-ol	24.388	2,681.3	1,646	1,035.3	C15H26O	0.41	Level 2*
26	Cadinol	24.592	2,718.4	1,653	1,065.4	C15H26O	8.86	Level 2*
27	3,5,6,7,8,8a-Hexahydro-4,8a-dimethyl-6-(prop-1-en-2-yl)naphthalen-2(1H)-one	25.173	2,828.8	1749	1,079.8	C15H22O	0.68	Level 2*
28	3-Ethenyl-3-methyl-2-(prop-1-en-2-yl)-6-(propan-2-yl)cyclohexan-1-ol	25.416	2,869.3	1,645	1,224.3	C15H26O	0.59	Level 2*
29	Bicyclo(3.3.1)nonane-2,6-dione	25.883	2,940.4	-	-	C9H12O2	0.38	Level 2*
30	2,2,7,7-Tetramethyltricyclo[6.2.1.0^2^,^6^]undec-4-en-3-one	25.969	2,952.7	-	-	C15H22O	1.44	Level 2*
31	1,4,9,9-Tetramethyl-5,6,7,8-tetrahydro-4H-3a,7-methanoazulene	26.331	3,004.4	1,472	1,532.4	C15H22	2.82	Level 2*
32	Ledene oxide-(II)	28.84	​	1,687	​	C15H24O	0.54	Level 2*

* Level 2: Probable - MS, match >90% + RI, deviation ±15.

**FIGURE 9:**
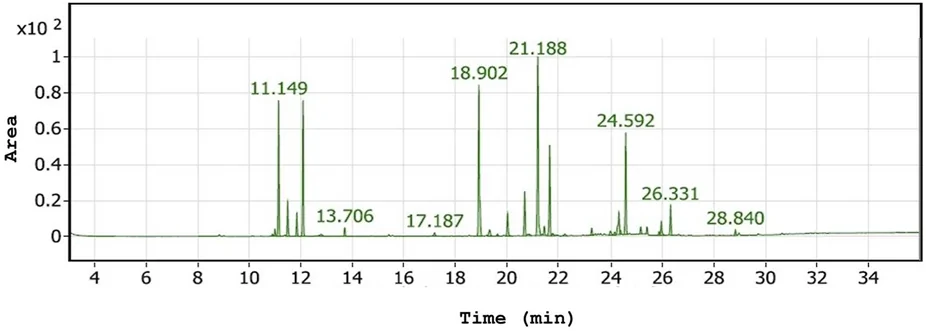
GC–MS chromatogram of EtOAc crude extract from endophytic *Cladosporium* sp.

Analysis of trimethylsilyl derivatization products of EtOAc extract from endophytic *Cladosporium* sp. by GC–MS revealed the presence of 14 compounds belonged to aliphatic and aromatic carboxylic acid, phenolic and fatty acids (Table 6; Figure 10). The fatty acids involving Oleic Acid, TMS derivative (51.48%) and 9,12-octadecadienoic acid, TMS derivative (linoleic acid, 37.06%) represent major compounds followed by palmitic Acid, TMS derivative (9.55). Other compounds were detected in very small amounts.

**TABLE 6:** GC-MS analysis of EtOAc crude extract from endophytic *Cladosporium* sp. derivatized using TMS.

Peak	Compound	RT (min)	RI calculated	RI literature*	Molecular formula	Similarity/Match score (%)	Relative abundance (%)	Identification level (MSI)
1	4-Methyl-2-oxovaleric acid, trimethylsilyl ester	4.554	1,150	N/A	C9H18O3Si	88	0.01	Level 3*
2	2-(Trimethylsilyloxy)hexanoic acid, trimethylsilyl ester	6.291	1,271.57	N/A	C12H28O3Si2	91	0.01	Level 2*
3	2-Phenylacetic acid, trimethylsilyl ester	6.878	1,315.06	1,320	C11H16O2Si	93	0.04	Level 2*
4	Butanedioic acid, bis(trimethylsilyl) ester	7.264	1,344.86	1,348	C10H22O4Si2	95	0.05	Level 2*
5	4-[(Trimethylsilyl)oxy]benzoic acid, trimethylsilyl ester	10.904	1,651.56	1,658	C13H22O3Si2	96	0.45	Level 2*
6	2-[4-[(Trimethylsilyl)oxy]phenyl]acetic acid, trimethylsilyl ester	11.047	1,664.71	1,672	C14H24O3Si2	94	0.04	Level 2*
7	3,4-Bis[(trimethylsilyl)oxy]benzoic acid, trimethylsilyl ester	13.035	1857.04	1865	C16H30O4Si3	92	0.18	Level 2*
8	Trimethylsilyl tetradecanoate	13.203	1873.94	1880	C17H36O2Si	94	0.05	Level 2*
9	Trimethylsilyl hexadecanoate (palmitic acid TMS)	15.141	2080.07	2085	C19H40O2Si	98	9.55	Level 2*
10	Trimethylsilyl (9Z,12Z)-octadeca-9,12-dienoate (linoleic acid TMS)	16.609	2,249.17	2,255	C21H40O2Si	98	37.06	Level 2*
11	Trimethylsilyl (9Z)-octadec-9-enoate (Oleic acid TMS)	16.676	2,257.14	2,262	C21H42O2Si	99	51.48	Level 2*
12	Bis(2-ethylhexyl) benzene-1,2-dicarboxylate (DEHP - contaminant)	19.385	2,566.4	2,575	C24H38O4	90	0.69	Level 2*
13	1-Oleoylglycerol bis(trimethylsilyl) ether (Monooleoylglycerol 2TMS)	20.988	2,804.57	2,812	C27H56O4Si2	89	0.19	Level 3*
14	Ergosta-5,7,22-trien-3-yl trimethylsilyl ether (Ergosterol TMS)	22.389	3,268.83	3,275	C31H52OSi	93	0.19	Level 2*

* Level 2: Probable - MS, match >90% + RI, deviation ±15; Level 3: Tentative - MS, match 85%–89% or absence of literature RI.

**FIGURE 10:**
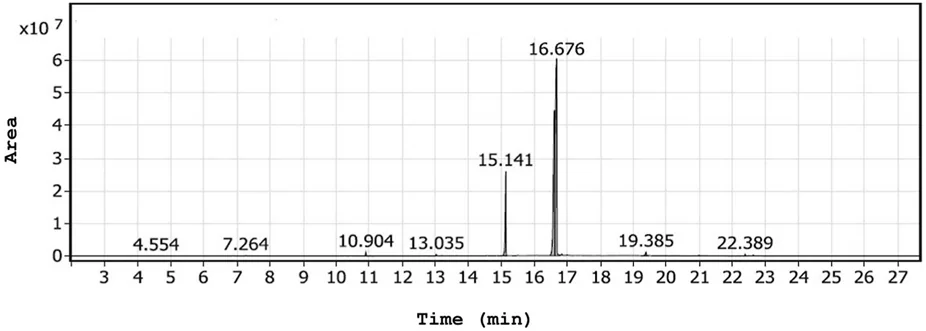
GC–MS chromatogram of TMS derivatized EtOAc crude extract from endophytic *Cladosporium* sp.

### 3.6 HPLC and LC-MS analysis

Analysis of the *Cladosporium* sp. EtOAc crude extract by HPLC-DAD against a panel of 18 phenolic acid and flavonoid reference standards resulted in the detection and quantification of seven phenolic compounds (Table 7; Figure 11). Chlorogenic acid was the most abundant quantified compound, at 7,276.29 μg/g, followed by caffeic acid at 4,829.88 μg/g. Gallic acid, syringic acid, methyl gallate, ferulic acid, and p-coumaric acid were quantified at 598.18, 253.10, 197.40, 180.95, and 119.22 μg/g, respectively. The HPLC-DAD method showed satisfactory linearity for the quantified analytes over the investigated concentration ranges. External calibration curves were constructed using the corresponding reference standards, and strong linear relationships between analyte concentration and detector response were obtained. The regression equations, calibration ranges, and coefficients of determination (R^2^) are presented in Table 8. In a complementary targeted LC-ESI-MS/MS analysis performed in MRM mode against 21 phenolic and flavonoid reference standards, 11 compounds were detected, whereas two additional analytes were detected at levels below their respective limits of quantification (LOQ) and were therefore not reliably quantified. Among the quantified analytes, 3,4-dihydroxybenzoic acid showed the highest concentration (3,563 μg/g), followed by caffeic acid (284.80 μg/g), ferulic acid (201.50 μg/g), p-coumaric acid (111.80 μg/g), ellagic acid (83.98 μg/g), gallic acid (57.90 μg/g), cinnamic acid (30.82 μg/g), and chlorogenic acid (13.92 μg/g). Syringic acid, quercetin, and kaempferol were detected at trace levels (Table 9; Figure 12).

**TABLE 7:** Retention time (Rt), area percentage, and concentration (μg/g) of HPLC-detected phenolic compounds from endophytic *Cladosporium* sp. EtOAc crude extract.

Compound name	Rt	Area	Conc. (µg/g)
Gallic acid	3.543	179.14	598.18
Chlorogenic acid	4.298	1,126.80	7,276.29
Catechin	4.500	0.00	0.00
Methyl gallate	5.504	75.56	197.40
Caffeic acid	5.871	1750.79	4,829.88
Syringic acid	6.431	85.79	253.10
Rutin	6.802	0.00	0.00
Ellagic acid	7.181	0.00	0.00
Coumaric acid	8.736	70.84	119.22
Vanillin	9.358	0.00	0.00
Ferulic acid	9.832	67.89	180.95
Naringenin	10.617	0.00	0.00
Rosmarinic acid	12.125	0.00	0.00
Daidzein	16.261	0.00	0.00
Quercetin	17.576	0.00	0.00
Cinnamic acid	19.676	0.00	0.00
Kaempferol	20.825	0.00	0.00
Hesperetin	21.494	0.00	0.00

**FIGURE 11:**
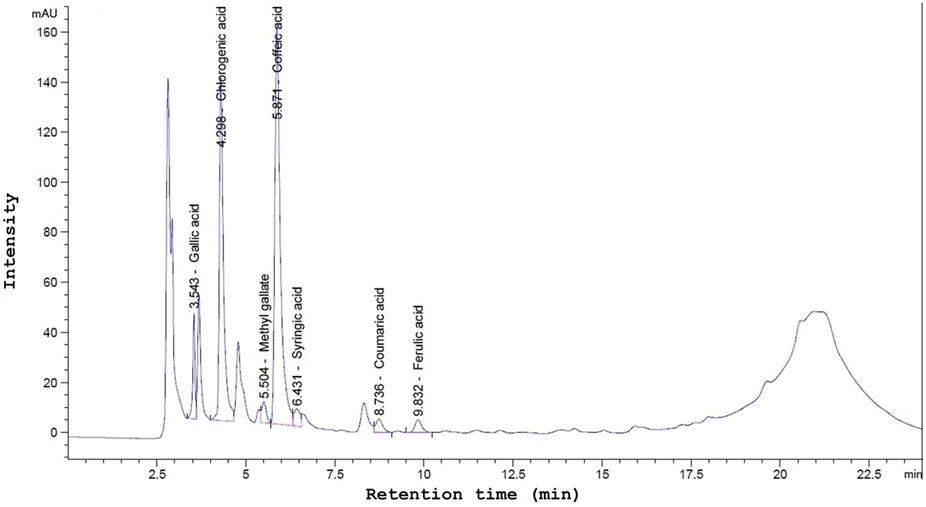
HPLC chromatogram of phenolic components of *Cladosporium* sp. EtOAc crude extract.

**TABLE 8:** LC-ESI-MS/MS characteristics and analytical validation parameters of the phenolic compounds determined by MRM.

Quantifier ion	Linear equation	*R* ^2^	LOD (ppm)	LOQ (ppm)
Gallic acid 168.9/124.9	y = 4.11887e6x - 6.18379e4	0.99329	0.01	0.05
Caffeic acid 178/135	y = 2.41216e7x - 1.18444e5	0.99545	0.01	0.05
Rutin 609/299.9	y = 1.07396e7x - 5.72596e4	0.99552	0.003	0.01
Coumaric acid 162.9/119	y = 6.09192e7x - 13,423.68072	0.99512	0.005	0.01
Vanillin 151/136	y = 2.06138e5x - 1725.80664	0.99641	0.01	0.05
Naringenin 271/151	y = 5.87334e4x - 305.65555	0.99487	0.01	0.05
Querectin 301/151	y = 3.38565e7x - 2.04299e5	0.99353	0.003	0.01
Ellagic acid 301/145	y = 1.46426e6x + 14,670.29707	0.99354	0.01	0.05
3.4-Dihydroxybenzoic acid 152.9/109	y = 4.58285e6x - 18,238.69092	0.99590	0.01	0.05
Hesperetin 301/136	y = 1.17669e7x - 24,851.92134	0.99719	0.005	0.01
Cinnamic acid 146.9/77	y = 7.94931e4x + 1,299.38454	0.99690	0.01	0.05
Methyl gallate 183/124	y = 9.40929e7x + 6.23680e4	0.99330	0.003	0.01
Kaempferol 284.7/93	y = 2.02145e6x - 3,973.66923	0.99510	0.005	0.01
Ferulic acid 192.8/133.9	y = 3.53847e6x + 961.90848	0.99370	0.005	0.01
Syringic acid 196.8/181.9	y = 1.41130e6x + 1,164.90208	0.99517	0.005	0.01
Apigenin 269/151	y = 1.72582e5x + 3,245.54558	0.99504	0.01	0.05
Catechin 288.8/109	y = 1.11120e6x + 1,543.33916	0.99255	0.005	0.01
Daidzein 253/132	y = 1.01615e7x + 3.25975e4	0.98587	0.005	0.01
Chlorogenic acid 353/191	y = 1.84167e7x - 4.60661e4	0.99576	0.003	0.01
Resveratrol 227/185	y = 3.27571e6x + 22,973.91253	0.98196	0.003	0.01
Rosmarinic acid 359.1/161	y = 3.04518e7x - 8.66246e4	0.99538	0.003	0.01

**TABLE 9:** Targeted LC-ESI-MS/MS determination of phenolic compounds in the EtOAc crude extract of endophytic *Cladosporium* sp.

Compound	Molecular formula	Precursor ion Q1 (m/z)	Product ion Q3 (m/z)	RT (min)	CE(V)	MRM transition
Gallic acid	C_7_H_6_O_5_	168.9	124.9	1.67	−30	168.9→124.9
3,4-Dihydroxybenzoic acid	C_7_H_6_O_4_	152.9	109.0	3.13	−40	152.9→109.0
Chlorogenic acid	C_16_H_18_O_9_	353.0	191.0	5.10	−35	353.0→191.0
Caffeic acid	C_9_H_8_O_4_	178.9	135.0	5.83	−22	178.9→135.0
Syringic acid	C_9_H_10_O_5_	196.9	122.8	6.23	−24	196.9→122.8
p-Coumaric acid	C_9_H_8_O_3_	162.9	119.0	7.70	−20	162.9→119.0
Ferulic acid	C_10_H_10_O_4_	192.8	133.9	8.89	−16	192.8→133.9
Ellagic acid	C_14_H_6_O_8_	301.0	145.0	8.97	−40	301.0→145.0
Cinnamic acid	C_9_H_8_O_2_	146.9	102.6	18.16	−17	146.9→102.6

**FIGURE 12:**
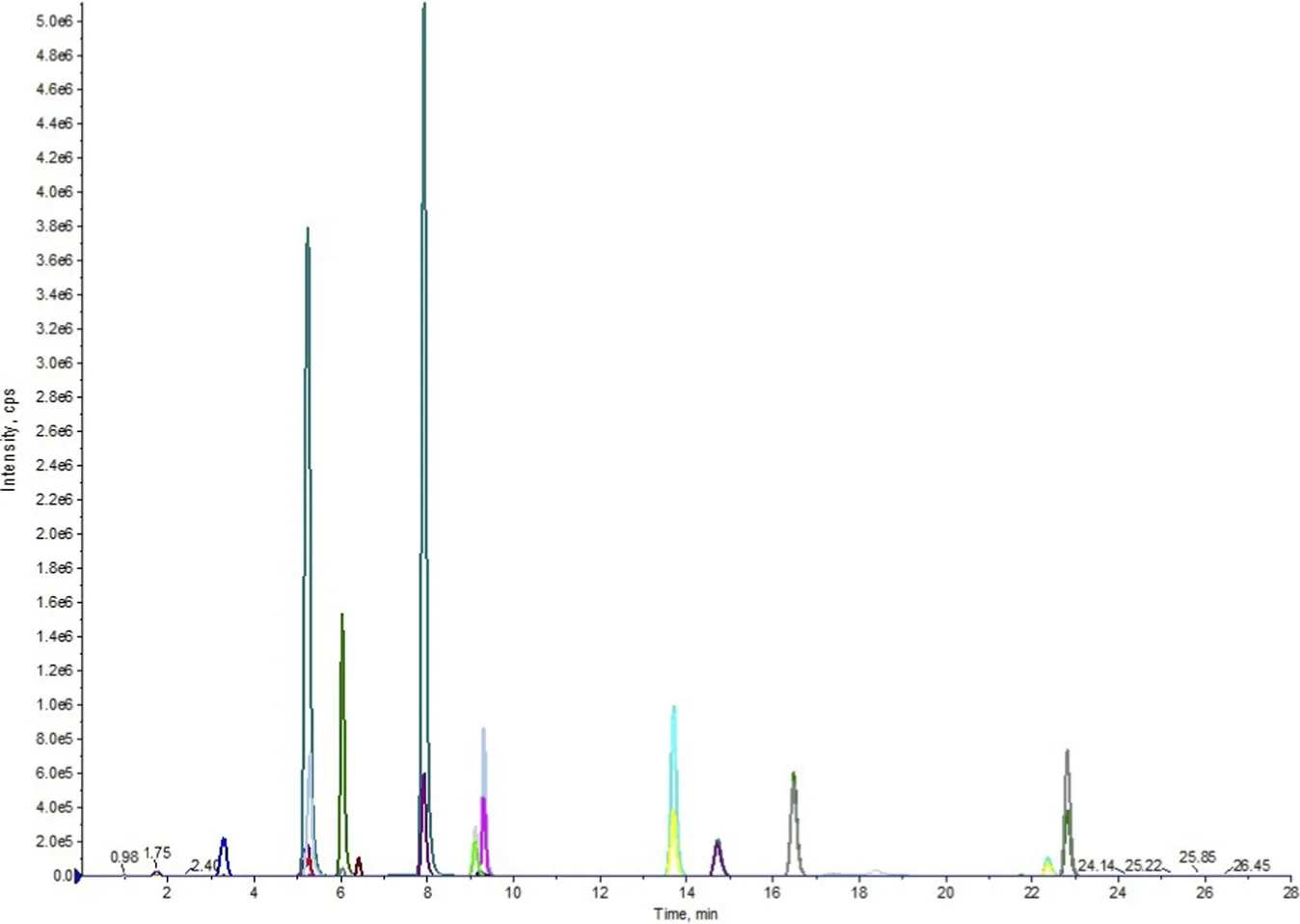
Extracted Ion Chromatogram (XIC) using LC-MS analysis of endophytic *Cladosporium* sp. EtOAc crude extract.

## 4 Discussion

This study reports, to our knowledge, the first endophytic association of *Cladosporium* sp. with *O. syriacum*, a medicinal plant recognized for its traditional and therapeutic value in Eastern Mediterranean. While *O. syriacum* has been extensively studied for its essential oils and phenolics, its endophytic mycobiome remains largely unexplored. The recovery of *Cladosporium* from surface-sterilized aerial tissues therefore expands the known host range for this genus and supports the hypothesis that medicinal Lamiaceae harbor chemically prolific endophytes that may contribute to the overall metabolomic diversity of the host-endophyte holobiont. Previous studies have reported antimicrobial, antioxidant, and cytotoxic activities among metabolites produced by endophytic fungi, suggesting that these microorganisms represent a valuable source of chemical diversity (Alma et al., 2003; Alwafa et al., 2021). No claim is made here that the endophyte directly contributes to the host’s traditional medicinal properties; such a link would require comparative metabolomics of endophyte-free vs. colonized plants and in planta localization studies. Taxonomic identification of isolate AUMC 15471 was based on ITS1-5.8S-ITS2 rDNA sequencing (533 bp). BLASTn analysis showed 100% identity to *Cladosporium* sp. isolate IISRBCCMF02 (PX459849) and *C. cladosporioides* strain F4 (PP188496), and 99.62% identity to *C. cladosporioides* strain DM11M1 (MT102827). ITS alone has limited discriminatory power within the *C. cladosporioides* species complex, which includes numerous cryptic species. The isolate is therefore conservatively retained as *Cladosporium* sp. AUMC 15471 pending multilocus phylogenetic analysis using actin (ACT), translation elongation factor 1-α (TEF1-α), and β-tubulin genes. This conservative approach avoids over-identification and aligns with current standards for *Cladosporium* taxonomy. The chemical profile of the EtOAc crude extract of *Cladosporium* sp. isolate reflects broad metabolic capacity rather than a single compound class. GC-MS of the unsaponified extract tentatively annotated 32 volatiles, mainly monoterpenes/terpenoids, sesquiterpenoids, and fatty alcohols. After TMS derivatization, 14 additional constituents, including aliphatic/aromatic acids and phenolics, were detected. HPLC-DAD (18 authentic standards) confirmed seven phenolics: gallic acid, chlorogenic acid, methyl gallate, caffeic acid, syringic acid, p-coumaric acid, and ferulic acid. LC-MS/MS (21 standards) detected 11 phenolics: gallic acid, 3,4-dihydroxybenzoic acid, chlorogenic acid, caffeic acid, syringic acid, p-coumaric acid, ferulic acid, ellagic acid, cinnamic acid, quercetin, and kaempferol.

Fragmentation patterns were consistent with reported diagnostic losses for phenolic acids (e.g., decarboxylation, [M-H-44]^-^; chlorogenic acid: 353.0→191.0 for quinic acid ion (Lyrio et al., 2025)). Concentration estimates differed between HPLC-DAD and LC-MS/MS for the same analytes (e.g., chlorogenic acid), reflecting differences in separation, detector response, ionization efficiency, and matrix effects. The two platforms were used complementarily for tentative identification, not as cross-validated quantitative methods, and direct quantitative comparison should be treated cautiously. Bis(2-ethylhexyl) phthalate (DEHP) was detected in the TMS-derivatized analysis. In the absence of procedural and matrix blanks, it cannot be assigned as an endogenous fungal metabolite and is more plausibly a plasticizer contaminant from solvent storage or handling. Similarly, several phenolics detected here (e.g., gallic, chlorogenic, caffeic, p-coumaric acid, quercetin) have been previously reported both in *O. syriacum* tissues (Afify et al., 2014), and in other *Cladosporium* endophytes (Qi et al., 2009; Singh et al., 2016; Salvatore et al., 2021). Accordingly, these compounds should not be assigned exclusively to fungal biosynthesis or host-derived metabolism without appropriate controls, such as uninoculated medium extracts, host-tissue controls, and comparative analyses of fungal cultures. The observed metabolite profile may therefore reflect fungal biosynthesis, transformation of host-associated compounds, or contributions from both sources, as suggested in recent studies of plant–endophyte metabolic interactions (Devi et al., 2023; Hashem et al., 2023). Future studies incorporating uninoculated rice-medium extracts, extraction/solvent blanks, independently processed *O. syriacum* tissue, and comparative fungal cultures analyzed under identical conditions would provide stronger evidence for assigning individual compounds to fungal biosynthesis.

In the present study, the EtOAc crude extract of *Cladosporium* sp. was evaluated for antibacterial activity against *Salmonella Typhimurium*, *E. coli* O157, *L. monocytogenes*, *Staphylococcus aureus*, *R. equi*, and *Campylobacter jejuni* at a single concentration of 1.5 mg/mL. Under the conditions of the assay, the extract produced variable levels of growth inhibition, with *S. aureus* showing the highest inhibition (67.37% ± 2.45%), followed by *C. jejuni* (59.39% ± 1.24%), *R. equi* (37.64% ± 1.33%), *S. Typhimurium* (31.53% ± 0.59%), *E. coli* O157 (24.28% ± 0.79%), and *Listeria monocytogenes* (12.65% ± 1.16%). These findings indicate that the crude extract exhibited measurable antibacterial effects at the tested concentration; however, the single-concentration design does not permit determination of antibacterial potency or calculation of minimum inhibitory concentration (MIC) or minimum bactericidal concentration (MBC). Therefore, the present results should not be interpreted as evidence of strong or potent antibacterial activity. Similarly, chloramphenicol (20 μg/mL) was included as a positive control to verify assay performance, but its activity cannot be directly compared with that of the crude extract because the two agents were tested at substantially different concentrations. The antibacterial activity should be considered a property of the crude EtOAc extract as a complex mixture rather than attributed to any individual metabolite detected during chemical profiling. The extract contained several phenolic compounds, including chlorogenic acid, caffeic acid, ferulic acid, cinnamic acid, and gallic acid, which have previously been reported to exhibit antibacterial effects against different bacterial species (Lou et al., 2011; Benbettaïeb et al., 2015; Kępa et al., 2018; Tian et al., 2022). Further studies using serial concentrations of the extract, concentration–response analysis, MIC and MBC determination, and appropriate independent biological replication are required to characterize its antibacterial potency more rigorously. Activity-guided fractionation followed by testing purified metabolites would also be necessary to determine whether individual compounds contribute to the observed antibacterial effects.

The antioxidant properties of the EtOAc crude extract of *Cladosporium* sp. were evaluated using three complementary *in vitro* assays: DPPH and ABTS radical-scavenging assays and the FRAP assay. The extract exhibited measurable radical-scavenging activity in both the DPPH and ABTS assays, with IC_50_ values of 230.21 and 183.70 μg/mL, respectively. Under the same experimental conditions, Trolox showed substantially lower IC_50_ values of 6.50 and 4.69 μg/mL, respectively, indicating considerably greater radical-scavenging potency of the reference antioxidant. Thus, the DPPH and ABTS activities of the fungal extract should be interpreted as measurable but relatively limited to moderate under the experimental conditions employed, rather than as evidence of high or potent antioxidant activity. The FRAP results further indicate reducing capacity of the crude extract; however, these chemical antioxidant assays provide evidence of *in vitro* chemical reactivity and should not be interpreted as demonstrating antioxidant efficacy in biological systems. The antioxidant response of the crude extract cannot be directly attributed to its total phenolic content or to any individual metabolite detected by chemical profiling, because no correlation analysis or compound-specific antioxidant testing was performed. In addition, the Folin–Ciocalteu-derived total phenolic content represents Folin–Ciocalteu-reactive reducing capacity and may be influenced by non-phenolic reducing substances. The observed antioxidant activity may therefore reflect the combined contribution and possible interactions of multiple constituents present in the crude extract. Previous studies have reported antioxidant properties for several compounds detected or potentially present in the extract (Hadidi et al., 2024; He et al., 2025; Zhang and Yu, 2026). Activity-guided fractionation, purification, and compound-specific antioxidant assays, together with appropriate concentration–response analyses, would be required to determine the individual or combined constituents contributing to the observed activity.

Cytotoxicity screening showed a concentration-dependent reduction in HepG2 cell viability, with an IC_50_ value of 93.43 μg/mL after treatment with the EtOAc crude extract. In contrast, an IC_50_ value was not reached in BNL cells within the tested concentration range (up to 100 μg/mL). These findings indicate that the extract affected HepG2 cell viability within the concentration range examined, whereas the concentration required to reduce BNL cell viability by 50% was not reached under the experimental conditions. However, these results should be interpreted cautiously and should not be considered sufficient evidence of selective cytotoxicity. In particular, the HepG2 IC_50_ value is close to the upper end of the tested concentration range, and accurate IC_50_ estimation would benefit from a broader concentration range with sufficient data points below and above the inflection region. Moreover, the comparison involves HepG2 cells of human origin and BNL cells of murine origin; therefore, differences in species and cellular characteristics may influence the observed responses and limit the interpretation of human tumor-versus-normal cell selectivity. Future studies should include an appropriate human normal hepatocyte model, such as a well-characterized human hepatic cell line or primary human hepatocytes, to provide a more relevant human-to-human comparison. Annexin V-FITC/PI flow-cytometric analysis further characterized the distribution of viable, apoptotic, and necrotic cell populations following treatment with the crude extract. After 48 h of exposure, the viable-cell population decreased from 95.82% ± 0.71% in the control to 84.40% ± 0.31% in treated cells, accompanied by increases in late apoptotic and necrotic populations. The late apoptotic population increased from 0.36% ± 0.15% in the control to 5.98% ± 0.06% following treatment, while the necrotic population increased from 2.20% ± 0.58% to 8.91% ± 0.21%. The early apoptotic population did not increase under the conditions examined. Thus, the Annexin V/PI results indicate an altered distribution of cell-death-associated populations, with increased late apoptosis and necrosis at the 48-h time point. Because only a single exposure time was evaluated, these data cannot establish a temporal progression from early to late apoptosis or distinguish definitively between primary necrosis and secondary necrosis. Time-course experiments and complementary molecular markers would be required to clarify the underlying cell-death processes.

Overall, the cytotoxicity and flow-cytometry findings describe the cellular response to the crude EtOAc extract as a complex mixture and do not establish the involvement of any specific metabolite or molecular pathway. Although some metabolites detected during chemical profiling have previously been associated with cytotoxic or other biological activities (Kimsa Dudek et al., 2022; Secme et al., 2023; Bozkurt et al., 2026). Activity-guided fractionation, purification, and compound-specific cytotoxicity assays, together with mechanistic studies, would be necessary to determine which constituents, if any, contribute to the observed cellular effects.

Compared with other *Cladosporium* isolates, the present strain exhibits a relatively high TPC (34.09 mg GAE/g extract) versus 0.76 mg/g reported for a marine algal-associated *C. cladosporioides* (Hulikere et al., 2016) and ∼25 mg GAE/g average for isolates from *Calotropis procera* and *Vernonia amygdalina* with DPPH IC_50_ of 1,142 and 480 μg/mL, respectively (Khiralla et al., 2016). Our DPPH IC_50_ (230 μg/mL) suggests somewhat stronger radical scavenging than those isolates, but such comparisons must be interpreted cautiously given differences in strain, host, culture conditions, extraction, and assay protocols. Overall, the antibacterial and cytotoxic profile is consistent with previous reports of bioactive *Cladosporium* endophytes, while extending them to a new host plant.

## 5 Conclusion

In conclusion, the endophytic *Cladosporium* sp. isolate obtained from *Origanum syriacum* exhibited a chemically diverse metabolite profile, as characterized through complementary GC–MS, HPLC-DAD, and LC–MS/MS analyses. The findings provide preliminary *in vitro* evidence that the EtOAc crude extract contains biologically active metabolites with antioxidant, antibacterial, and cytotoxic activities. However, these bioactivities should be attributed to the EtOAc crude extract as a whole, and direct associations between specific detected metabolites and the observed biological effects cannot be established from the present data. Although the identified metabolites are not individually novel, their occurrence in an endophytic *Cladosporium* isolate associated with *O. syriacum* contributes valuable information to the understanding of the chemical diversity of this fungal–plant system. The observed biological activities, particularly against HepG2 cells and selected foodborne pathogens, indicate that this isolate may represent a promising source of bioactive secondary metabolites deserving further investigation. Nevertheless, the current findings remain exploratory and should be interpreted within the limitations of an *in vitro* study. Additional research is required to identify the compounds responsible for the observed activities through activity-guided fractionation, isolation, and structural characterization of active constituents. Further studies should also include mechanistic investigations, analytical standardization, safety and toxicity assessments, and *in vivo* validation before any potential pharmaceutical, nutraceutical, or food-preservation applications can be considered.

## Data Availability

The ITS rDNA sequence of Cladosporium sp. isolate AUMC 15471 has been deposited in the GenBank database under accession number OM854799. All other data generated or analyzed during this study are included in this published article and its Supplementary Material. Additional information may be obtained from the corresponding author upon reasonable request.
