# Supplementary material for: Metabolite profiling and in vitro biological activities of an endophytic Cladosporium sp. AUMC 15471 isolated from Origanum syriacum L
Source: Front Pharmacol. 2026 Oct 23;17:1950031. doi: 10.3389/fphar.2026.1950031 (PMC13739699; doi:10.3389/fphar.2026.1950031)
Supplement: Supplementary file 1 [file Table1.docx]

# Supplementary Table S1. Complete concentration-response data and Selectivity Index

**Table S4A. BNL mouse normal liver cells – raw data from NAWAH Report R-SO31344 (SRB, 72 h, 5 concentrations, triplicate).**

| Conc (µg/mL) | Rep1 OD 540nm | Rep2 OD | Rep3 OD | Mean Viability % | SD | n |
| --- | --- | --- | --- | --- | --- | --- |
| 0 (Control) | 2.5742 | 2.5923 | 2.5552 | 100 | 0 | 3 |
| 0.01 | 2.5987 | 2.6082 | 2.5158 | 100.01 | 1.63 | 3 |
| 0.1 | 2.4784 | 2.3843 | 2.3422 | 93.22 | 2.24 | 3 |
| 1 | 2.4274 | 2.3726 | 2.3563 | 92.59 | 1.20 | 3 |
| 10 | 2.3620 | 2.3122 | 2.3129 | 90.37 | 0.92 | 3 |
| 100 | 2.3025 | 2.3012 | 2.3112 | 89.42 | 0.17 | 3 |
| Blank | 0.0324 | 0.0325 | 0.0303 | - | - | - |
| DOX IC50 |  |  |  | 0.07 µg/mL |  |  |

**Table S1B. HepG2 human hepatocellular carcinoma – preliminary IC50 data (SRB, 48 h). Complete curve to be extended to 400 µg/mL in future work.**

| Conc (µg/mL) | Mean Viability %* | SD | n | Note |
| --- | --- | --- | --- | --- |
| 0 | 100 | 0 | 3 | Vehicle control |
| 0.01 | 99.5 | 1.5 | 3 |  |
| 0.1 | 98.2 | 2.0 | 3 |  |
| 1 | 95.1 | 1.8 | 3 |  |
| 10 | 88.4 | 2.1 | 3 |  |
| 100 | 47.2 | 3.2 | 3 | Close to IC50 |
| IC50 | 93.43 µg/mL (preliminary, wide CI) |  |  | Close to top dose – low precision |

**Table S1C. Selectivity Index (SI) calculation.**

| Cell line | Species | Type | IC50 (µg/mL) | SI = IC50(normal)/IC50(cancer) | Interpretation |
| --- | --- | --- | --- | --- | --- |
| BNL | Mouse | Normal liver | >100 (89.42% viability at 100 µg/mL, not reached) | **>1.07 (lower-bound)** | No significant cytotoxicity up to 100 µg/mL; does NOT demonstrate safety |
| HepG2 | Human | Hepatocellular carcinoma | 93.43 (preliminary, close to upper limit) | 1.0 (reference) | Low potency, wide CI – requires extended range |
| DOX (positive control) | - | - | 0.07 (BNL) | - | Assay validation – highly cytotoxic |

**Notes:** SI >3 is generally considered moderate selectivity, SI >10 high selectivity (Badisa et al., 2009). Current SI >1.07 indicates lack of selectivity.
