# Supplementary material for: Metabolite profiling and in vitro biological activities of an endophytic Cladosporium sp. AUMC 15471 isolated from Origanum syriacum L
Source: Front Pharmacol. 2026 Oct 23;17:1950031. doi: 10.3389/fphar.2026.1950031 (PMC13739699; doi:10.3389/fphar.2026.1950031)
Supplement: Supplementary file 2 [file Table2.docx]

LOCUS	OM854799	533 bp	DNA	linear	PLN 28-FEB-2022

DEFINITION Cladosporium sp. strain AUMC 15471 internal transcribed

spacer 1, partial sequence; 5.8S ribosomal RNA gene and internal

transcribed spacer 2, complete sequence; and large subunit

ribosomal RNA gene, partial sequence.

ACCESSION	OM854799

VERSION	OM854799

KEYWORDS	.

SOURCE	Cladosporium sp.

ORGANISM Cladosporium sp.

Eukaryota; Fungi; Dikarya; Ascomycota; Pezizomycotina;

Dothideomycetes; Dothideomycetidae; Cladosporiales;

Cladosporiaceae; Cladosporium.

REFERENCE	1 (bases 1 to 533)

AUTHORS	Hassane,A.M.

TITLE	Direct Submission

JOURNAL	Submitted (27-FEB-2022) Assiut University Mycological Centre,

Assiut University, Faculty of Science, Assiut, Assiut 71511, Egypt

COMMENT	##Assembly-Data-START##

Sequencing Technology :: Sanger dideoxy sequencing

##Assembly-Data-END##

FEATURES	Location/Qualifiers

source	1..533

/organism="Cladosporium sp.

/mol_type="genomic DNA"

/strain="AUMC 15471"

/isolation_source="Endophyte"

/host="Origanum syriacum"

/db_xref="taxon:29917"

/country="Egypt: Assiut"

/collection_date="12-Mar-2021"

/collected_by="Abdalla M. Hassane"

misc_RNA	<1..>533

/note="contains internal transcribed spacer 1, 5.8S

ribosomal RNA, internal transcribed spacer 2, and large

subunit ribosomal RNA"

ORIGIN

1 aggtgaaaac tgcggaggga tcattacaag tgaccccggt ttaccaccgg gatgttcata

61 accctttgtt gtccgactct gttgcctccg gggcgaccct gccttcgggc gggggctccg

121 ggtggacact tcaaactctt gcgtaacttt gcagtctgag taaacttaat taataaatta

181 aaacttttaa caacggatct cttggttctg gcatcgatga agaacgcagc gaaatgcgat

241 aagtaatgtg aattgcagaa ttcagtgaat catcgaatct ttgaacgcac attgcgcccc

301 ctggtattcc ggggggcatg cctgttcgag cgtcatttca ccactcaagc ctcgcttggt

361 attgggcaac gcggtccgcc gcgtgcctca aatcgaccgg ctgggtcttc tgtcccctaa

421 gcgttgtgga aactattcgc taaagggtgt tcgggaggct acgccgtaaa acaaccccat

481 ttctaaggtt gacctcggat caggtaggga tacccgctga acttaagcat atc

//
